# NADcapPro and circNC: methods for accurate profiling of NAD and non-canonical RNA caps in eukaryotes

**DOI:** 10.1038/s42003-023-04774-6

**Published:** 2023-04-13

**Authors:** Sunny Sharma, Jun Yang, John Favate, Premal Shah, Megerditch Kiledjian

**Affiliations:** 1 Department of Cell Biology and Neurosciences, Rutgers University, Piscataway, NJ USA; 2 Department of Genetics, Rutgers University, Piscataway, NJ USA

**Keywords:** RNA metabolism, RNA

## Abstract

Accurate identification of NAD-capped RNAs is essential for delineating their generation and biological function. Previous transcriptome-wide methods used to classify NAD-capped RNAs in eukaryotes contain inherent limitations that have hindered the accurate identification of NAD caps from eukaryotic RNAs. In this study, we introduce two orthogonal methods to identify NAD-capped RNAs more precisely. The first, NADcapPro, uses copper-free click chemistry and the second is an intramolecular ligation-based RNA circularization, circNC. Together, these methods resolve the limitations of previous methods and allowed us to discover unforeseen features of NAD-capped RNAs in budding yeast. Contrary to previous reports, we find that 1) cellular NAD-RNAs can be full-length and polyadenylated transcripts, 2) transcription start sites for NAD-capped and canonical m^7^G-capped RNAs can be different, and 3) NAD caps can be added subsequent to transcription initiation. Moreover, we uncovered a dichotomy of NAD-RNAs in translation where they are detected with mitochondrial ribosomes but minimally on cytoplasmic ribosomes indicating their propensity to be translated in mitochondria.

## Abstract (web summary)

Two methods NADcapPro and circNC are presented for precise identification of NAD-caps and non-canonical caps in eukaryotic RNAs.

## Introduction

The presence of modified nucleosides is a characteristic feature of the majority of cellular RNAs. Modified nucleosides generally ascend from the chemical modification of a genetically encoded nucleoside. One exception is the addition of an N7-methylguanosine to the 5ʹ end of select eukaryotic RNAs transcribed by RNA polymerase II referred to as “m^7^G cap”^1^. Historically only eukaryotic transcripts were believed to contain 5ʹ end modifications since the original analyses of bacterial RNA composition did not detect modification on the 5′ end^2,3^. However, seminal work in the late 1970s^4^ and early 2000^5^ established that nucleotide metabolites, by virtue of their adenosine nucleotide moieties (NAD and FAD), could be utilized by *E. coli* and T7 RNA polymerases as initiating nucleotides for RNA synthesis in vitro. The initial evidence that NAD caps are indeed incorporated into RNA in cells was provided by mass spectrometry (LC-MS) in bacteria^6^. Nonetheless, due to the inability of MS analysis to provide the sequence context, the physiological role of these noncanonical caps remained elusive.

To assess the significance of the presence of the NAD cap in cellular RNA, a chemo-enzymatic and Next Generation Sequencing (NGS) based method, known as NAD captureSeq (NADcapSeq), was introduced in 2015^7^. This method not only corroborated the presence of NAD-capped RNAs (hereafter referred to as NAD-RNA) in bacteria but also divulged the identity of the transcripts. NADcapSeq transpired to be a decisive advance in assessing the function of these caps in RNA metabolism. Subsequent NADcapSeq analyses of yeast^8^, plant^9^, human^10^, and archaeal RNA^11,12^ have led to the identification of a significant number of transcripts with a NAD cap and have established the presence of the NAD cap as a 5′ non-canonical cap in all three domains of life.

NADcapSeq leverages the unique feature of Adenosine Diphosphate Ribosyl Cyclase (ADPRC) from *Aplysia californica* to replace nicotinamide with alkynyl alcohol, in a transglycosylation reaction^13^. The ‘clickable’ transglycosylation product is next biotinylated by a copper-catalyzed azide-alkyne cycloaddition (CuAAC)^7^. After enriching the biotin-linked RNAs using streptavidin beads, the RNAs are subjected to NGS-based RNA Seq analysis to identify the NAD-RNAs. Although NADcapSeq is a robust method for cataloging NAD-RNAs, recent studies have highlighted two of its key limitations; (a) the dependence on the use of copper ions as a metal catalyst for the *Huisgen cycloaddition* reaction^14^, and (b) ADPRC promiscuity^15,16^. Metal ions, including copper, can induce RNA fragmentation, which could hinder the detection of low-abundant RNAs, and manifest a bias toward shorter fragmented reads from the 5′ termini of relatively abundant RNA^17^. A further limitation of profiling NAD-capped RNAs by NADcapSeq in eukaryotes involves the residual promiscuity of the ADPRC enzyme on the m^7^G-capped RNAs^16^ (hereafter referred to as m^7^G-RNA). Depletion of m^7^G-RNA by an anti-m^7^G cap antibody has been used as one strategy to minimize the level of m^7^G-RNA in the NAD-RNA population^16^. Although this is an improvement, additional approaches to streamline NAD cap detection are indispensable for the rapid and accurate profiling of the NAD-RNAs in eukaryotes. Furthermore, the current lack of high-resolution mapping of the NAD position in eukaryotes precludes mechanistic determination of NAD cap addition.

Here we report two methods, NAD cap profiling (NADcapPro) Seq, and intramolecular circularization of Noncanonical-Capped (circNC) RNA. NADcapPro Seq builds on the recently reported use of copper-free Strain-Promoted Azide-Alkyne Cycloaddition (SPAAC)^14^ to identify NAD-capped transcripts in *Arabidopsis*^16^, which depended on m^7^G cap antibody depletion to minimize contaminating m^7^G-RNAs. NADcapPro Seq circumvents the antibody depletion by coupling the robust m^7^G decapping activity of Dcs1^18^ prior to the SPAAC reaction. Dcs1 abolishes the unfavorable residual enzymatic activity of ADPRC towards canonical m^7^G cap. Moreover, direct visualization of the NAD-capped RNAs was possible with streptavidin-conjugated IRDye. A second method, circNC, provides an independent mechanism to detect non-canonical capped RNAs at the nucleotide level of precision. Analysis of NAD-capped RNAs initially detected by NADcapPro subjected to circNC enabled us to extrapolate that NAD caps are not only incorporated as noncanonical initiating nucleotides in place of ATP but, can also be post-transcriptionally added in eukaryotic cells. Furthermore, NAD caps were found to be associated with mitochondrial ribosomes, raising the possibility that they can be translated in mitochondria.

## Results

### Development of NADcapPro and visualization of NAD-RNAs

**Fig. 1:**
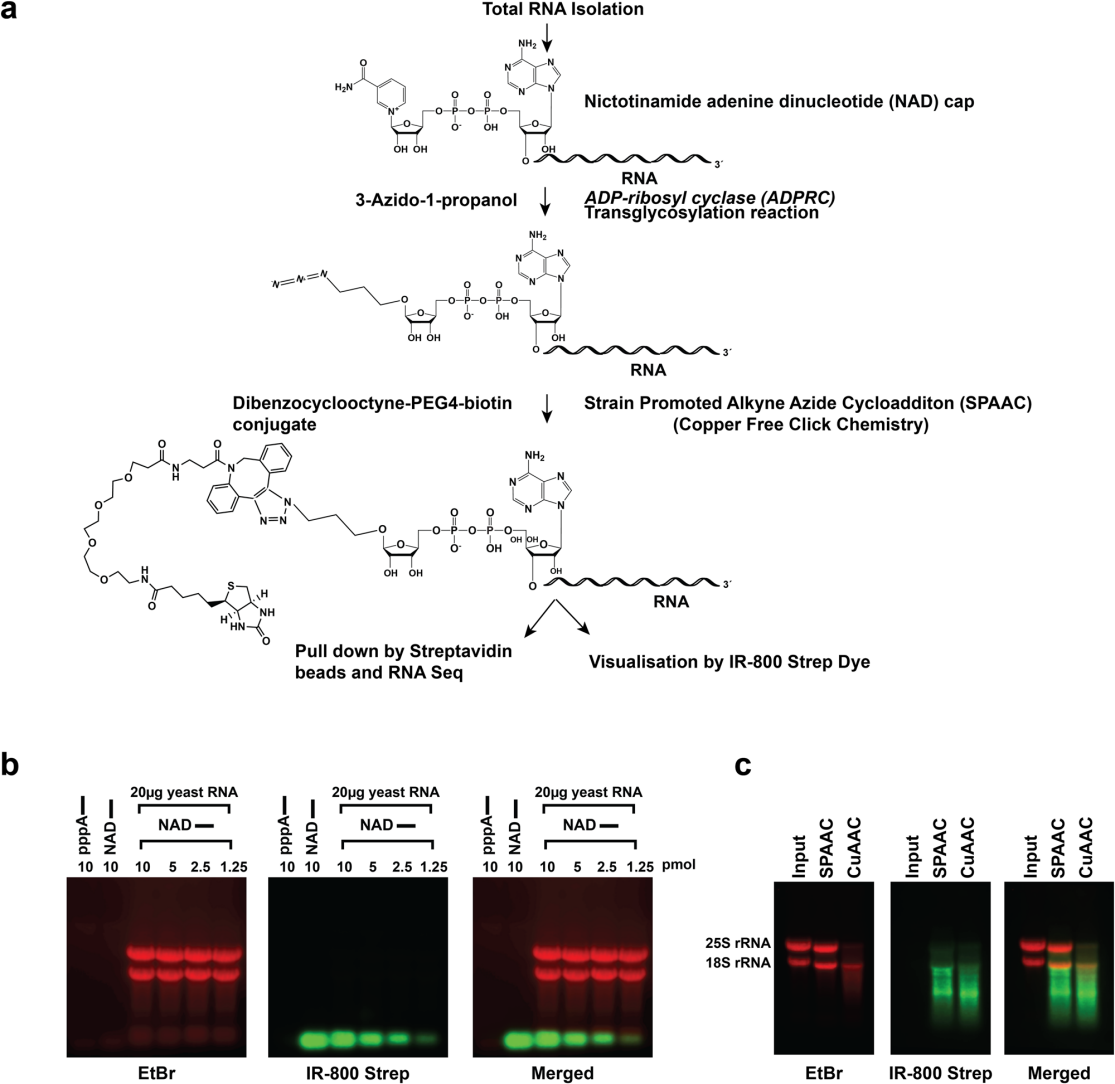
SPAAC chemistry is superior to CuAAC for characterizing NAD-capped RNAs. **a** Schematic illustration of the SPAAC reaction to profile NAD-capped RNAs. **b** Ten picomoles of 40 nts in vitro transcribed RNA with a 5′ triphosphate (pppA) or 5′ NAD cap were subjected to the SPAAC reaction. The RNAs were resolved on an agarose gel at the indicated concentration either without or with the addition of 20 µg of background total yeast RNA and transferred onto a Nitrocellulose membrane. The biotin-conjugated RNAs (NAD) were visualized by near-infrared (IR) IRDye® 800CW streptavidin-based detection using Odyssey Fc (Li-Cor Biosciences). The NAD cap or uncapped RNA is represented by the NAD of pppA followed by a line that denotes the RNA. **c** Twenty micrograms of total yeast RNA were subjected to SPAAC and CuAAC reactions and the RNAs were detected using IRDye® 800CW. The CuAAC reactions led to RNA degradation while the SPAAC reactions minimally affect the integrity of the RNA.

A key limitation of the NADcapSeq method used previously for detecting NAD-RNAs is the use of copper ions as a metal catalyst for the *Huisgen cycloaddition* reaction^14^. An alternative approach based on copper-free chemistry, the strain-promoted azide-alkyne cycloaddition (SPAAC) reaction^19^ was recently reported^16^. Instead of using 4-pentyn-1-ol (NAD capSeq), 3-azido-1-propanol is used for the ADPRC-catalyzed transglycosylation reaction (Fig. 1a) where an azide moiety instead of an alkyne is generated to replace nicotinamide, which would then become amenable to the SPAAC reaction^19^. The azide residue introduced upon transglycosylation can be subjected to the autocatalytic alkyne moiety of biotin-PEG4-Dibenzylcyclooctyne (biotin-PEG4-DBCO) to obviate the need of using copper ions (Fig. 1a and Supplementary Fig. 1a).

We expanded on the use of the previously reported SPAAC approach^16^ with two important modifications including direct visualization of the NAD-RNAs and elimination of contaminating m^7^G-capped RNAs. In vitro transcribed NAD-RNAs and triphosphate (pppA) RNAs were subjected to the SPAAC reaction and the biotin-conjugated RNA (corresponding to NAD-RNA); was visualized by near-infrared (IR) IRDye® 800CW streptavidin-based detection. As observed in Fig. 1b, IRDye® 800CW streptavidin enables direct visualization of biotinylated NAD-RNAs and importantly validated the feasibility of the SPAAC reaction for detecting NAD-RNAs. This was further verified by using the same NAD-RNA substrate subjected to NAD decapping (deNADding) by Rai1 treatment prior to the SPAAC reaction which eliminated the signal (Supplementary Fig. 1b). Rai1 is a well-characterized deNADding enzyme that removes the intact NAD moiety without degrading the subsequent RNA^10^. Moreover, the sensitivity of IR dye for the detection of NAD-RNAs is in the fmol range. SPAAC reactions with different concentrations of in vitro transcribed NAD-RNAs demonstrated that biotin-conjugated RNAs can be detected by the IR dye at levels as low as 15.6 fmol (Supplementary Fig. 1c). Importantly as previously reported^20^, negligible levels of background autofluorescence and a higher signal-to-noise ratio is apparent for the IR dye approach. Next, to assess the advantage of SPAAC over CuAAC^7^ in terms of preserving the RNA integrity, total RNA isolated from yeast cells was subjected independently to SPAAC and CuAAC reactions. As expected, using previously published conditions for the CuAAC reactions led to RNA degradation while the SPAAC reactions minimally compromised RNA integrity (Fig. 1c). However, whether further optimization of the CuAAC reaction may also minimize RNA degradation is not clear.

**Fig. 2:**
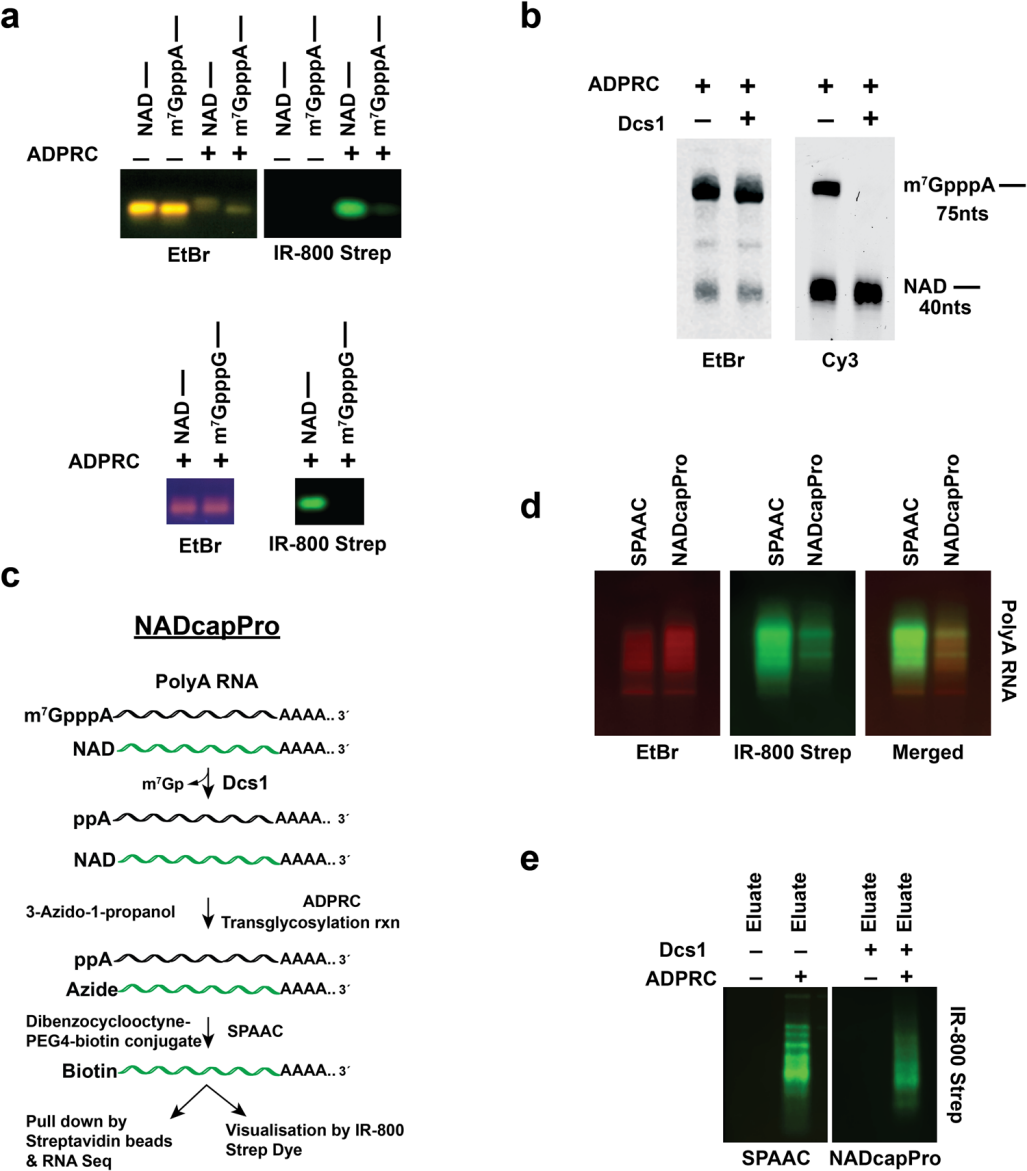
ADPRC cross-reactivity with m^7^GpppA capped RNAs and NADcapPro as a superior method to resolve the inherent limitations of previous methods. **a** Ten picomoles of 40nts in vitro transcribed NAD-, m^7^GpppA- and m^7^GpppG-capped RNA were subjected to the SPAAC reaction and visualized using IRDye® 800CW. Detection of biotinylated RNA analyzed by SPAAC revealed residual activity only on m^7^GpppA-capped RNA. **b** In vitro transcribed m^7^GpppA-RNA (75nts) with and without Dcs1 and 40nts of NAD-capped RNA were mixed at molar ratios of (50:1) in a SPAAC reaction. The RNAs were visualized directly in the gel. ADPRC exhibited a noticeable activity towards m^7^GpppA capped RNA in the absence of Dcs1 treatment. Treatment of the RNA mixture with Dcs1 explicitly abolished the reactivity of the APRC towards the m^7^GpppA-capped RNA without compromising the detection of NAD-RNA. **c** Schematic illustration of NADcapPro protocol used in the present study. **d** Comparison of the SPAAC and NADcapPro reactions using endogenous polyA+ RNA isolated from yeast were resolved by agarose gel electrophoresis and visualized using IRDye® 800CW. A substantial reduction in NAD-RNA signal is apparent in the NADcapPro lanes compared to the SPAAC lanes, consistent with the selective removal of m^7^G-capped RNA detection. **e** PolyA+ RNA (15 µg) was subjected to SPAAC and NADcapPro. The affinity-purified biotinylated RNA corresponding to NAD-capped RNA was eluted from streptavidin beads and visualized using IRDye® 800CW as in (**d**).

Current approaches to scoring NAD-capped RNAs in eukaryotes are limited by the detection of background residual m^7^G-capped RNA^10,16^ which conflates the two populations of caps. To begin optimizing eukaryotic NAD-RNA isolation, we delineated the nature of this background more systematically by assessing the residual activity of ADPRC on canonical-capped RNAs, especially m^7^GpppA and m^7^GpppG (account for the majority of transcription start site (TSS) base^21^). Consistent with previous reports^10,16^, detection by IR dye of biotinylated RNA analyzed by SPAAC revealed residual activity on m^7^GpppA capped RNAs (Fig. 2a, and Supplementary Fig. 7). Interestingly, this was not observed with m^7^GpppG RNAs demonstrating a level of specificity previously unknown for adenosine as the first transcribed nucleotide. With the recent mass spectrometry-based estimates of relatively higher m^7^GpppA-capped RNA to NAD-RNA^9^ even minor levels of detection could skew NAD cap detection.

To profile bona fide NAD-RNAs more accurately in eukaryotes, we developed a modified SPAAC approach to enzymatically remove m^7^G-RNAs from the reaction. We leveraged the selective activity of the yeast scavenger decapping enzyme Dcs1^18^ and its robust pyrophosphatase activity on m^7^G-capped RNA that hydrolyzes the phosphodiester bond between the gamma and beta phosphates to release a 5′-diphosphate-RNA (ppRNA) and a 7-methyl-guanosine monophosphate^18^ prior to SPAAC modification. We termed this approach NAD cap profiling (NADcapPro).

To assess the feasibility of NADcapPro, a mixture of in vitro transcribed 75 nts m^7^GpppA-capped RNA and 40 nts NAD-RNA at molar ratios of ~50:1 was used in the absence or presence of Dcs1 treatment. Visualization of the RNA following the SPAAC reaction was carried out with DBCO-Cy3 to visualize the clicked RNAs, avoiding the prerequisite to blot the RNA onto a membrane. As shown in Fig. 2b, ADPRC indeed displayed a noticeable activity towards m^7^GpppA-capped RNA in the absence of Dcs1 treatment with robust detection of the m^7^GpppA-capped RNA. In contrast, treating the RNA mixture with Dsc1 specifically abolished the reactivity of the ADPRC towards the m^7^GpppA capped RNA without affecting the detection of NAD-RNAs, demonstrating the feasibility of NADcapPro for identifying NAD-RNAs in eukaryotes. This was next validated by using endogenous polyA^+^ RNAs from WT cells treated with and without Dcs1 prior to the SPAAC reaction (Fig. 2c). Visualization of the RNAs revealed a substantial reduction in IR dye signal in the NADcapPro lanes compared to the SPAAC lanes, consistent with the selective removal of m^7^G RNA detection (Fig. 2d). In addition, the RNA subjected to NADcapPro can be retained and eluted from streptavidin beads (Fig. 2e) to enable subsequent analysis.

### Transcriptome-wide mapping of NAD-RNAs in yeast

**Fig. 3:**
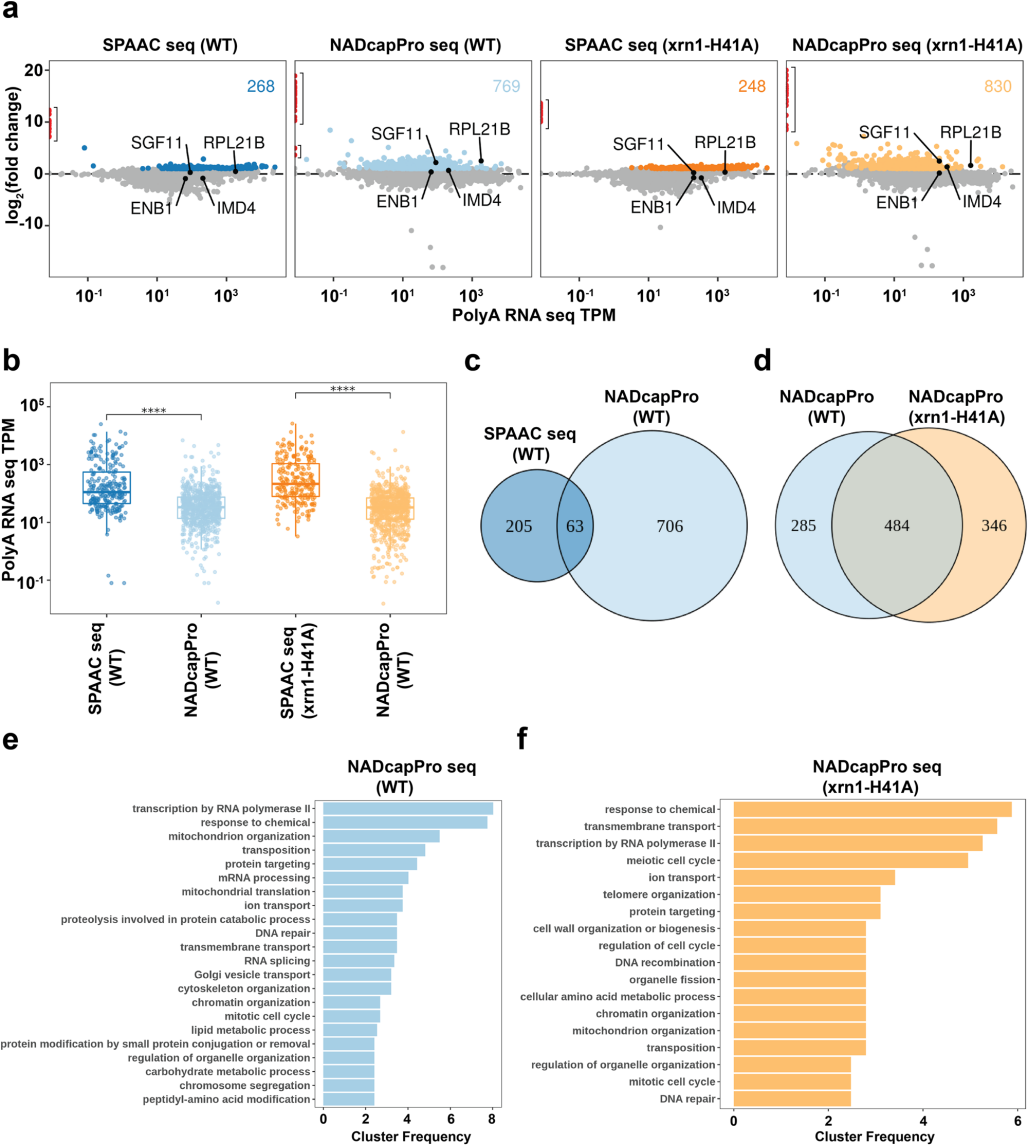
NADcapPro Seq. **a** The relationship between RNA abundance and fold-change in the experimental condition is indicated at the top of each panel. The x-axis indicates RNA abundance from RNAseq derived from WT (blues) or xrn1 mutant (oranges) cells. The y-axis indicates the DESeq2 fold change. Colored points indicate a fold-change log_2_ ≥ 1 and q-value ≤ 0.01. The number in the upper right indicates the number of genes meeting these criteria. **b** NADcapPro can capture low abundance transcripts. The distribution of average TPMs for the colored genes in (**a**) is shown. ****p < 0.0001 for a one-sided t-test testing if the NADcapPro distributions are less than the SPAAC distributions. Venn diagram overlap of the indicated gene sets in WT, NADcapPro and Xrn1-targeted NAD-RNAs are represented in (**c**) and (**d**) respectively. Gene ontology categories for the indicated gene sets in WT, NADcapPro (**e**) and Xrn1-targeted NAD-RNAs (**f**). All source data are contained in the GSE217259 repository.

To demonstrate the feasibility of NADcapPro at the genomic scale, we generated standard RNA-seq, SPAAC-seq and NADcapPro-seq datasets derived from a WT strain and a strain disrupted for deNADding activity of a prominent deNADding protein Xrn1 (*xrn1-H41A*)^22^. Differential expression analyses using DESeq2^23^ identified transcripts enriched in SPAAC or NADcapPro relative to standard RNA-seq. We classified a gene as potentially capped if it was enriched by at least twofold over the standard RNA-seq at a q-value ≤0.01 (Fig. 3a). Based on these two metrics, SPAAC-NAD-seq identified 268 transcripts, whereas NADcapPro seq identified 769 NAD-capped transcripts in WT cells and 830 NAD-capped transcripts in the *xrn1-H41A* cells. The *xrn1-H41A* protein is deNADding deficient yet retains the 5′-3′ exoribonuclease activity^22^ and is expected to have a higher number of NAD-RNAs. However, the differential is modest since yeast harbor additional deNADding enzymes^10,17^ and Xrn1 appears to have a restricted NAD-RNA target population^22^. In addition, although a higher number of NAD-RNAs evident with NADcapPro relative to SPAAC may appear counterintuitive, it is the removal of the abundant contaminant m^7^G-capped transcripts that enables the detection of these low-abundance NAD-RNAs. For example, four transcripts that were scored below the significance threshold in SPAAC (RPL21B, ENB1, IMD4, and SGF11) were identified as NAD-RNAs by NADcapPro (Fig. 3a) and further validated to be NAD-capped by an independent approach (see below).

Additional support for the specificity of NADcapPro was evident upon analysis of the identified transcript’s abundance. Whereas SPAAC is only able to detect highly expressed NAD-RNAs, NADcapPro can detect NAD-RNAs with lower expression. This is illustrated in Fig. 3b, where the Transcripts Per Kilobase Million (TPM) distribution of potentially capped genes from NADcapPro are lower than the SPAAC distribution. Interestingly as shown in Fig. 3c, only 63 transcripts (~8% of the total NAD-capped transcripts) overlapped between SPAAC and NADcapPro seq, a strong indication that most transcripts identified by SPAAC seq corresponded to the relatively higher abundance of m^7^GpppA-capped transcripts which in turn likely impeded the capture of the moderately abundant NAD transcripts. A comparison of the number of NAD transcripts responsive to the robust Xrn1 deNADding activity (*xrn1-H41A*) to that of WT cells revealed 346 transcripts that were directly responsive to Xrn1 deNADding (Fig. 3d) while the remaining transcripts appear to be regulated by deNADding enzymes other than Xrn1^10,17^. This is in contrast to our initial use of the CuAAC approach to identify NAD transcripts responsive to Xrn1 that only identified the abundant mitochondrial NAD transcripts with confidence^22^. Collectively, these results demonstrate that NADcapPro seq is more robust and adept at capturing high and low-abundance NAD transcripts that are otherwise not detected with the standard NAD-RNA isolation approaches.

Gene Ontology (GO) term analysis was carried out to ascertain the potential biological processes the refined list of NAD-RNAs may be involved in (Figs. 3e and 3f). Investigation of the 706 transcripts and 346 transcripts from the WT and *xrn1-H41A* mutant along with the transcripts in common to both (Supplementary Fig. 2) respectively yielded a diverse array of pathways. In addition to the chemical and transcriptional machinery of RNA pol II, a high cluster frequency was observed for nuclear-encoded mitochondrial genes involved in both mitochondrial organization and mitochondrial translation in the WT pool. This is consistent with NAD being an important cofactor for RNA transcription and mitochondrial function. The Xrn1-responsive NAD-RNAs also were similarly involved in chemical and transcriptional responses, in addition to transmembrane transport and the meiotic cell cycle. The most striking GO term among the top pathways that exhibited a relatively greater cluster frequency was “Transposition” encompassing the process involved in mediating the movement of discrete segments of DNA between nonhomologous sites. Intriguingly, these transcripts were undetected with standard RNA sequencing but are enriched by more than log_2_ > 10 by NADcapPro (highlighted in red in Fig. 3a) suggesting that transcripts belonging to this GO term are predominantly NAD-capped. The importance for NAD capping of these transcripts (Supplementary Table 1) remains to be determined; however, it is tempting to speculate that NAD capping of these RNA may be a major contributor to their low abundance consistent with NAD caps promoting RNA decay^10^.

### Non-canonical caps can be added following transcription initiation

**Fig. 4:**
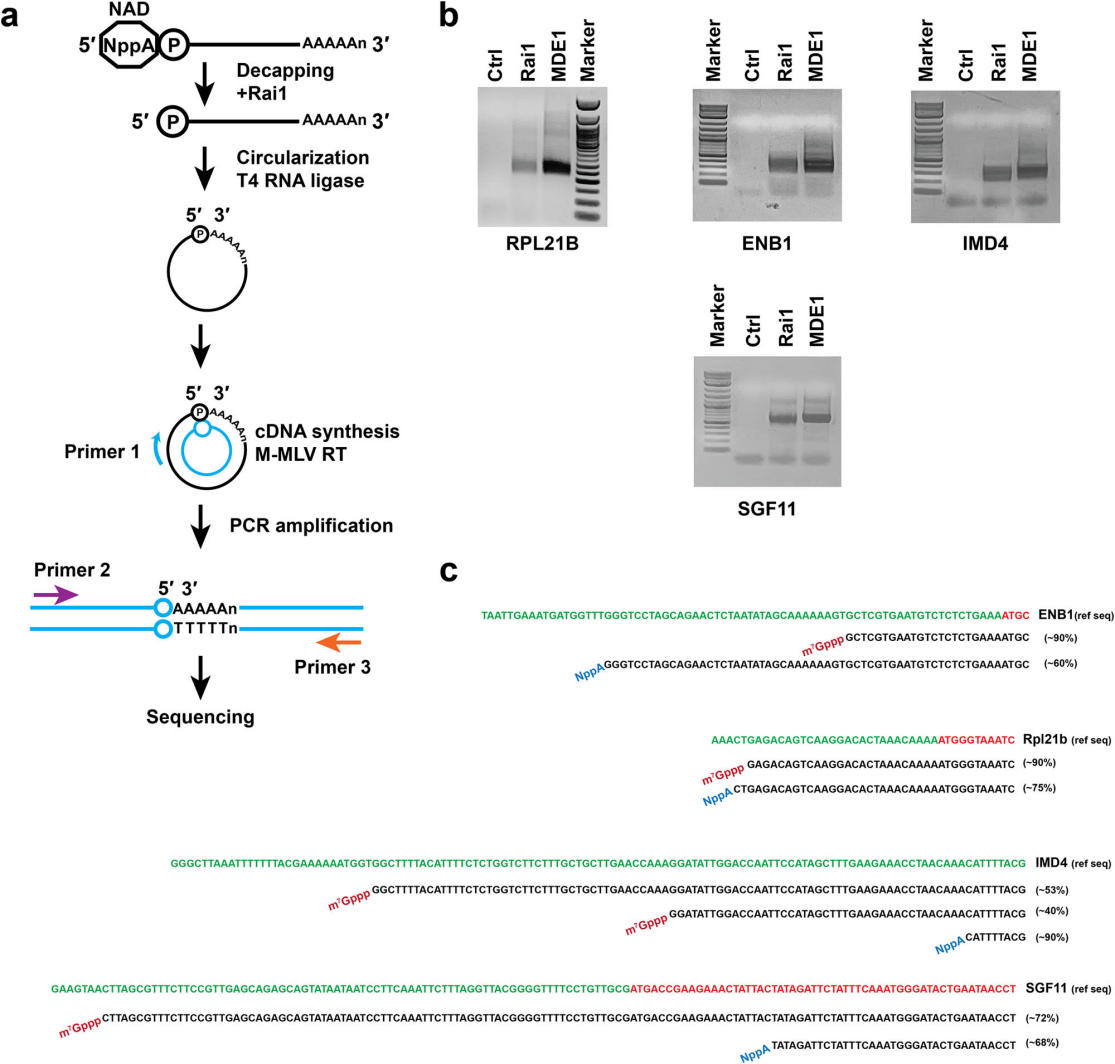
circNC as an orthogonal method for non-canonical-capped RNA detection. **a** Schematic illustration of the circNC method. **b** Transcript-specific PCR of validated NAD-capped RNAs subjected to circularization following Rai1 treatment (NAD-RNA) or MDE treatment (m^7^G-RNA) as illustrated in (**a**) and resolved on 2% TAE-agarose gel. **c** Sequence alignment of the Rai1 and MDE-treated samples derived from next-generation sequencing of the PCR products generated from the nuclear-encoded transcripts are shown. The alignment represents the most prevalent annotated reads spanning the 5′-3′ junction. The percentage of reads corresponding to the depicted sequences are shown on the right of each sequence. The green color of the reference sequence represents the 5′ UTR while the red region denotes the open reading frame starting with the ATG initiation methionine sequence.

The current dogma postulates that NAD caps are incorporated during transcription initiation as noncanonical nucleotides in place of ATP as the first transcribed nucleotide^24,25^. Although there is in vitro evidence in support of this hypothesis, cell-based evidence is lacking in eukaryotes. To directly address whether NAD caps can be added by a transcription initiation independent mechanism we developed an approach to map the precise NAD metabolite nucleotide addition site within transcripts validated by NADcapPro to possess NAD caps. We built on two previously reported methodologies, CapZymeSeq^26^ and RNA end circularization^27^ to develop an orthogonal approach which we termed intramolecular circularization of Noncanonical-Capped RNA, circNC (Fig. 4a). Here we leveraged the robust in vitro deNADding activity of Rai1, which removes the intact NAD moiety from the 5′ end of an RNA to generate a 5′ monophosphate RNA^10^. Importantly, Rai1 does not hydrolyze m^7^G-capped transcripts^10^ (Supplementary Fig. 3a). The resulting monophosphate RNA is intramolecularly circularized using T4 RNA ligase to produce a circular RNA with the 5′ untranslated regions (UTR) fused to the 3′ end polyA tail followed by the 3′ UTR region. The use of transcript-specific primers enables cDNA synthesis followed by specific PCR amplification across the junction.

CircNC was used to validate four of the nuclear-encoded NAD transcripts identified by NADcapPro from Fig. 3a: RPL21B, ENB1, IMD4, and SGF11 (Supplementary Fig. 4a). The use of circNC on these yeast NAD-capped mRNAs yielded the expected PCR product following Rai1 deNADding (Fig. 4b). The size of the band was comparable to that generated when the RNA was decapped with the m^7^G-capped mRNA decapping enzyme (MDE) (Fig. 4b) which is unable to hydrolyze NAD-capped RNA (Supplementary Fig. 3a) or triphosphate RNA (Supplementary Fig. 3b). To verify the specific amplicons and assess the sequence context of the respective NAD caps, the PCR products were subjected to next-generation sequencing (Supplementary Fig. 4b). The sequence analyses of the Rai1 and MDE-treated samples are shown in Fig. 4c and Supplementary Data 1. Since transcription initiation sites can be variable, we focused on the most prominent transcripts for each cap and each mRNA. Two important differences are apparent when comparing the canonical m^7^G and NAD transcripts. First, the predominant 5′ ends of the m^7^G-capped and NAD-capped transcripts are distinct and they do not share a common transcription start site. Second, two of the four NAD transcripts analyzed do not start with an adenosine moiety in the coding strand. The importance of this latter observation is that non-canonical caps are believed to be incorporated onto the 5′ ends of prokaryotic mRNA by the incorporation of NAD rather than ATP as the first transcribed nucleotide during transcription initiation^24^. By extension, this has also been presumed to be true in eukaryotic cells. Such a mechanism necessitates the presence of an adenosine nucleotide in the coding strand of the DNA. However, as apparent with the NAD cap positions in ENB1 and SGF11, corresponding adenosine is not evident at the NAD cap addition site in the reference coding sequence. In the case of RPL21B and IMD4, the position of the NAD does contain an A residue consistent with a co-transcriptional NAD addition. We conclude NAD cap addition can be carried out by a post-transcriptional initiation process of NAD capping. Furthermore, the analyzed transcripts were all polyadenylated, indicating that NAD transcripts are intact full-length mRNAs, contrary to previous reports^17^. The 5′ end skewed representation of NAD transcripts in previous reports was likely a byproduct of CuAAC-mediated RNA fragmentation during the NAD capture.

### Nuclear-encoded NAD-RNAs are likely not translated

Since nuclear encoded NAD-RNAs have intact 5′ and 3′ UTR and are polyadenylated akin to canonical capped transcripts, we next tested if the NAD transcripts are translated. Initial reports using a transfected NAD-capped RNA in mammalian cells did not detect any appreciable level of translation^10^. However, definitive interpretations of these data were complicated by the fact that an exogenous transcript was used rather than an endogenous NAD-RNA. Support for the translation of NAD-RNAs was subsequently reported in *Arabidopsis thaliana* where NAD-capped transcripts were detected within cytoplasmic polysomes^9^. However, as these studies relied on the NADcapSeq approach, it was not clear whether the detected RNAs were indeed NAD-capped as previously reported^16^.

**Fig. 5:**
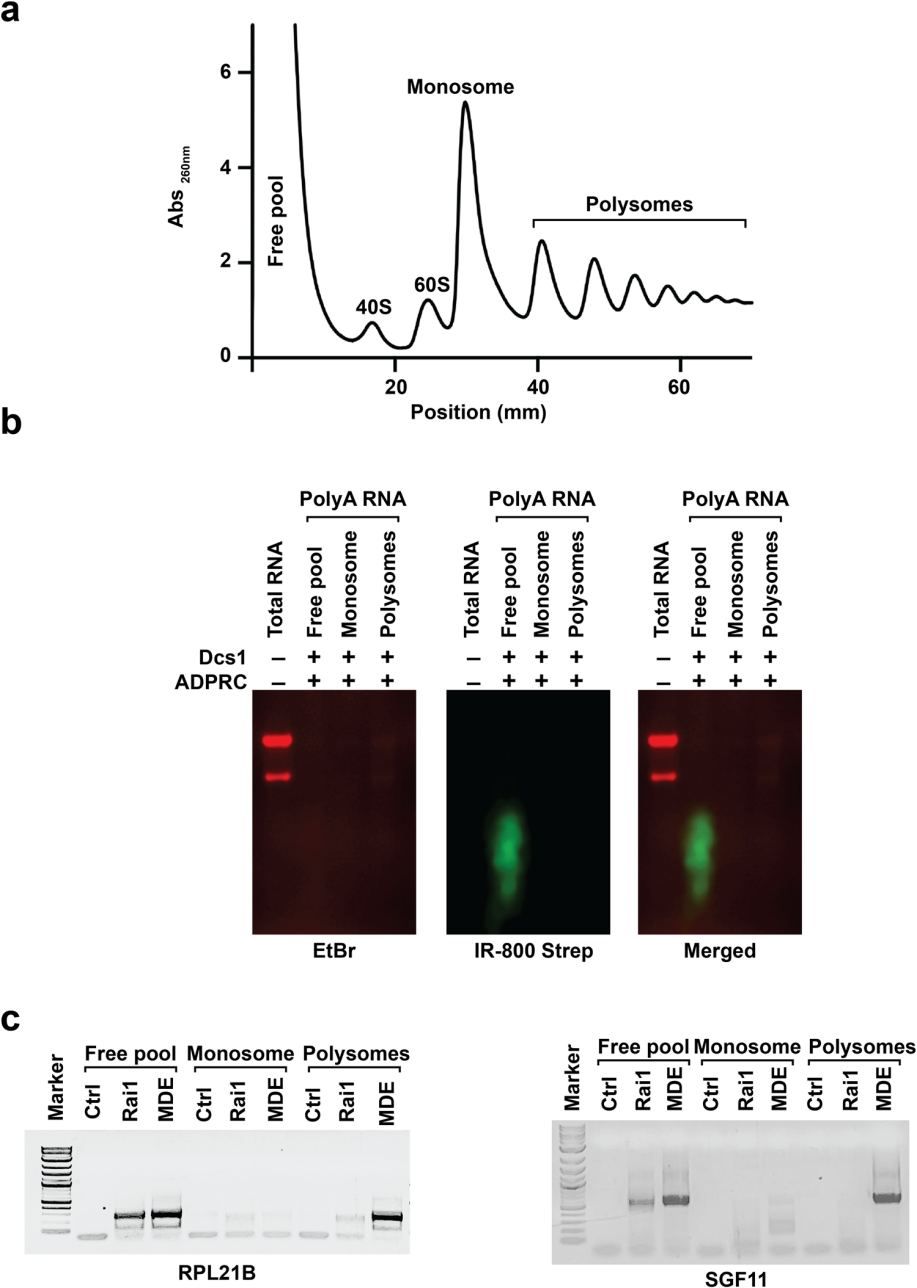
Nuclear-encoded NAD-capped RNAs are not translated. Polysome gradient profile of WT cells. Sucrose-density gradient ultracentrifugation was used to fractionate the free pool, ribosomal subunits, monosome, and polysomes. **a** Optical density profiles (Abs_260nm_) of polysome gradient are shown. PolyA+ RNA was isolated from the free pool, monosome, and merged polysome fractions and analyzed by NADcapPro. **b** NAD-RNAs were visualized using IR dye. Noticeably, NAD-capped RNAs were exclusively detected in the free pool representative of the fraction of polyA+ RNAs not engaged in translation. **c** PolyA+ RNA from the polysome gradients was subjected to circNC analysis and transcript-specific PCR of the RPL21B and SGF11 circularised RNAs are shown. The Rai1 and MDE treated lanes represent NAD- and m^7^G-capped RNAs respectively. The Ctrl is RNA that was not treated with either Rai1 or MDE enzymes. PCR products were resolved on a 1.5% TAE-agarose gel.

To assess whether NAD-RNA can associate with actively translating pools of RNA, we isolated RNAs from polysome gradients and subjected them to NADcapPro (Fig. 5a). PolyA+ RNA was isolated from the free pool, monosome, and merged polysome fractions and analyzed by NADcapPro. NAD-RNAs were visualized using IR dye. Intriguingly, NAD-RNAs were predominantly detected in the free pool representing the fraction of cellular polyA+ RNAs that are not engaged in translation and minimally in the translationally active polysome fractions (Fig. 5b and Supplementary Fig. 8). To further validate this observation, polyA+ RNAs isolated from the same fractions were subjected to circNC analysis. As shown in Fig. 5c, circNC of two NAD transcripts, RPL21B and SGF11, further corroborated the NADcapPro results and revealed that most NAD transcripts are excluded from translationally active ribosomes and are likely not engaged in translation.

### Mitochondrial NAD-RNAs are associated with translating ribosomes

**Fig. 6:**
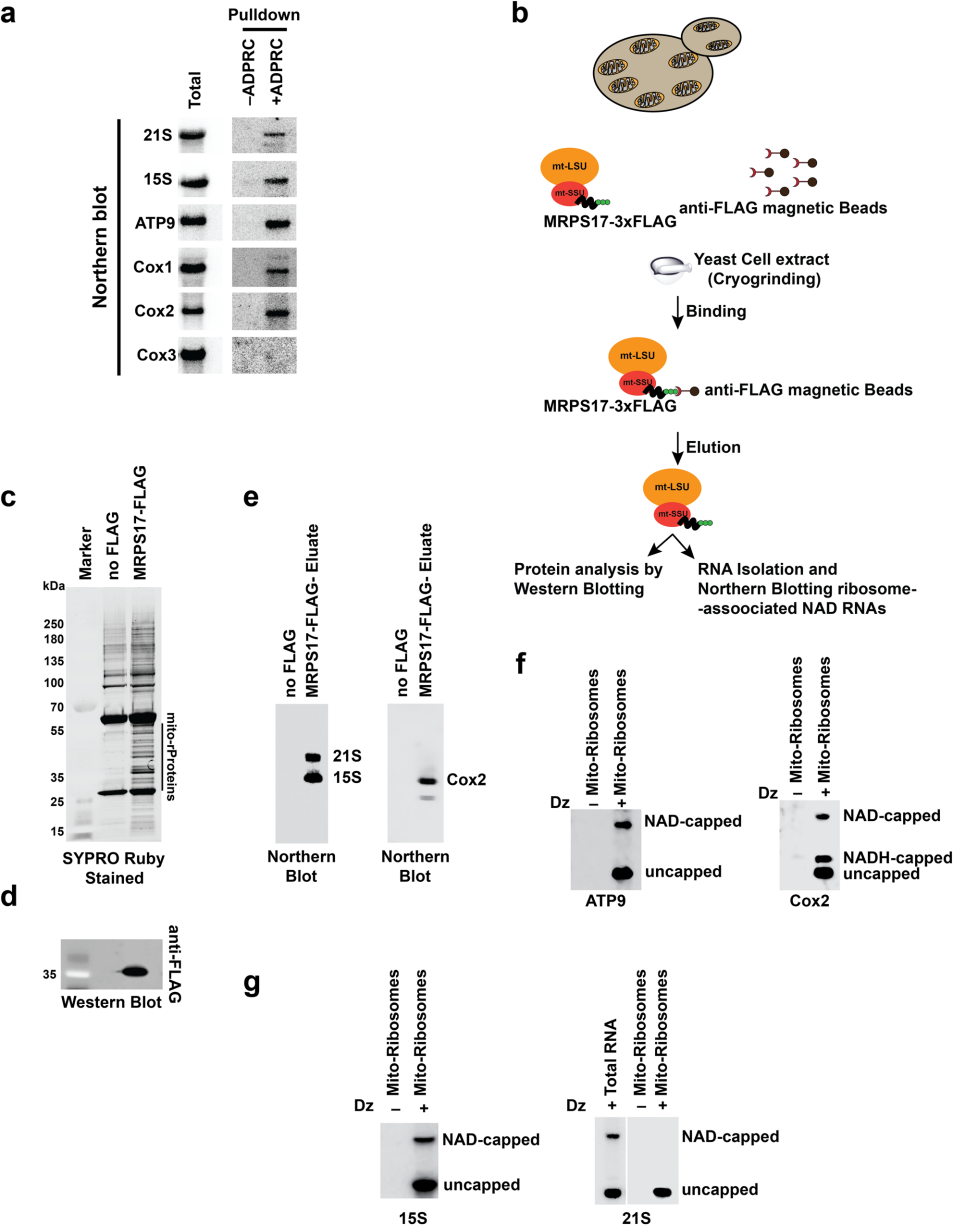
Mitochondrial NAD-RNAs are associated with a translationally active pool of mitoribosomes. Comprehensive NAD-capped RNA profile of yeast mitochondrial transcripts by SPAAC analysis. **a** Northern blot analysis of the RNA eluates from the affinity-purified NAD-capped RNA by SPAAC. **b** Schematic illustration of the strategy used to isolate the translationally active pool of ribosomes as explained recently by^28^. **c** Eluates from MRPS17-FLAG- IP were loaded onto a 4–12% Bis-Tris gel and stained with SYPRO Ruby. The no FLAG affinity purification was used as a control. **d** Western blot analysis with an anti-FLAG-tag antibody for the validation of the presence of MRPS17-FLAG-tagged protein. **e** Northern Blot analysis of RNA isolated from the MRPS17-FLAG- IP eluates. Northern Blot analysis of DNAzyme-generated 5′-end-containing subfragments of the RNA isolated from the MRPS17-FLAG- IP eluates resolved on 0.3% 3-acrylamidophenylboronic acid 10% PAGE gel and detected with the ^32^P labeled transcript-specific probes of the indicated mRNAs in (**f**) and ribosomal RNAs in (**g**).

The initial report identifying NAD-capped transcripts in *S.cerevisiae* indicated a predominance of NAD caps on mitochondrial encoded transcripts^8^, and two, Cox2 and 21 S rRNA, were subsequently validated by an independent method^22,24^. Yeast mitochondria encode 8 major protein-coding genes – Cox1, Cox2, Cox3, COB, SCE1, ATP6, ATP9, and VAR1 along with two mitochondrial ribosomal RNAs -15 S and 21 S rRNA, and 24 tRNAs. Since mitochondrial transcripts lack a polyA tail, they were not included in our above NADcapPro analysis. We next set out to establish a comprehensive NAD-RNA profile of protein-coding and rRNA yeast mitochondrial transcripts by SPAAC analysis of total RNA isolated from purified mitochondria and assessed NAD transcripts by Northern blotting using the RNA eluates from the affinity purified NAD-RNAs. The lack of m^7^G caps on mitochondrial transcripts precluded the need to employ NADcapPro. Only 5 NAD mitochondrially encoded transcripts were detected to be NAD-capped (Fig. 6a, and Supplementary Fig. 9). Three corresponded to protein-coding transcripts –Cox1, Cox2, and ATP9, and two were the non-coding mitochondrial ribosomal RNAs – 21 S rRNA and 15 S rRNA (Fig. 6a, and Supplementary Fig. 9). We further validated NAD capping of ATP9, Cox1, and 15 S rRNA using DNAzymes (Supplementary Fig. 5, and Supplementary Fig. 8).

The presence of protein-coding mitochondrial mRNAs possessing a NAD cap prompted us to test whether these transcripts are found in actively translating polysomes. To address this question, mitochondrial ribosomes were affinity purified using a recently established protocol^28^. A FLAG-tagged nuclear-encoded mitochondrial small subunit ribosomal protein MRPS17 was used as bait to affinity purify mitochondrial ribosomes (Fig. 6c, d, and Supplementary Fig. 10) under conditions that optimize immunoprecipitation of membrane-associated mitoribosomes whilst retaining subunit association^28^ (Fig. 6b). Northern Blot analysis of the immunopurified products demonstrated that intact mitoribosomes consisting of both 15 S and 21 S rRNA subunits as well as an mRNA (Cox2) are affinity-purified (Fig. 6e), consistent with actively translating ribosomes as previously reported^28^. To assess the presence of NAD transcripts in this translationally active ribosome pool, boronate affinity electrophoresis^29,30^ was used to analyze two of the protein-coding transcripts, Cox2 and ATP9, and two non-coding rRNA (15 S and 21 S) in more detail. This method renders direct visualization of both NAD-capped and uncapped RNA populations by specifically impeding the mobility of NAD-RNAs in the gel due to the transient formation of diesters between immobilized boronic acid and the ribose moiety^29,30^. DNAzyme-mediated RNA cleavage was used to generate 5′-end containing sub-fragments of defined length^22^, and a Northern blot analysis with the ^32^P labeled transcript-specific probes was used to corroborate the presence of NAD-capped transcripts. Remarkably, unlike nuclear-encoded transcripts, both ATP9 and Cox2 NAD mRNAs are associated with the translationally active mitochondrial ribosomes (Fig. 6f, and supplementary fig. 11). Interestingly, the Cox2 mRNA also possesses NADH-capped RNA as previously reported^24^, while this was not detected on any of the other mitochondrial transcripts. The rationale of this distinction is not clear. An additional intriguing observation was that only one of the NAD-capped derivatives of the ribosomal RNAs (15 S rRNA) is detected in the mature ribosome, but not the 21 S rRNA (Fig. 6g, and supplementary fig. 11) even though both can be NAD-capped (Fig. 6a). Collectively, our data demonstrate that, unlike cytoplasmic NAD-RNAs, NAD-capped mitochondrial mRNAs are associated with translating ribosomes and may be translated.

## Discussion

The presence of NAD as a 5′ noncanonical cap in cellular RNAs has been established in various organisms^7,8,10,16^. To understand the biological function of 5′ NAD caps in cellular physiology, the identification of cellular NAD-RNAs is imperative. The original method introduced to catalog transcriptome-wide NAD-RNAs in different organisms-NADcapSeq was instrumental in understanding the nature of NAD caps in prokaryotic RNA despite the limitation of using copper-based click reaction (CuAAC) that favored 5′ RNA fragments. However, more pertinently, its extrapolation into analyzing eukaryotic mRNAs with m^7^G caps was problematic due to the promiscuity of the ADPRC reaction. To address this issue, Hu et al^16^ reported the use of copper-free SPAAC-mediated NAD-RNA isolation and proposed the use of m^7^G-capped RNA depletion to enrich for NAD-RNA. Herein we expanded on the use of copper-free SPAAC-mediated NAD-RNA isolation and coupled the SPAAC reaction with a more robust enzymatic approach to eliminate m^7^G-capped RNAs to accurately profile NAD-RNAs in eukaryotes and further leveraged the highly sensitive IRDye® 800CW streptavidin-based detection to visualize NAD-RNAs at concentrations as low as 15 fmol. Of note, a recent report also used an enzymatic approach to minimize m^7^G capped RNA within the NAD population by eluting biotin-conjugated NAD-RNAs with a NAD decapping enzyme^31^.

NADcapPro incorporates the capacity of ADPRC to use an azide for the transglycosylation reaction through a copper-free click-chemistry-based SPAAC reaction that maintains RNA integrity. It also addresses the cross-reactivity of ADPRC with the canonical m^7^GpppA-caps in eukaryotes by exploiting the robust m^7^G decapping activity of yeast Dcs1^18^. Dcs1 treatment of RNA prior to the ADPRC reaction eliminates ADPRC reactivity towards the m^7^GpppA-capped RNAs (Fig. 2b) and enables affinity purification of true NAD-RNAs along with the identification of a relatively higher number of NAD-RNAs that previously remained obscure. Importantly, we also provide an orthogonal circNC method to validate and expand on the NADcapPro approach. CircNC is a sensitive approach to identifying RNAs harboring a non-canonical cap and enables precision mapping of the cap addition site to the nucleotide level as well as the precise 3′ termini of the RNA. While NAD caps are the primary focus of our study, it is worth noting that Rai1 can also decap FAD^32^ and dephospho CoA caps^32^. Therefore, although the RNAs used in the circNC analysis consisted of RNAs validated by NADcapPro to possess an NAD cap, the formal possibility remains that a mixture of non-canonical caps may be detected. Future optimization with Rai1 mutants that can distinguish between distinct non-canonical caps will further enhance the methodology.

Our approaches to NAD-RNA isolation and visualization enabled a refined analysis of the NAD nucleotide metabolite-capped class of RNAs. First, the length and state of NAD-RNAs have been confounded by different results of whether NAD-RNAs can be full-length or not^10,17^. circNC by virtue of detecting the polyA tail at the junction of the circularized RNA enables an assessment that NAD-RNAs can be full-length (Fig. 4a, c). Second, the 5′ ends of m^7^G-capped RNAs and their corresponding NAD-capped transcripts were different and did not map to the same start sites. Notably, although they did contain relatively similar polyA tail lengths, two of the four NAD-capped transcripts analyzed, SGF11 and IMD4, contained relatively longer 3ʹUTRs than their m^7^G-capped counterpart transcripts (Supplementary Data 1). Third, a comparison of the NAD cap addition site to the genomic sequence revealed NAD caps that did not align with corresponding adenosine in the genomic sequence indicating that the NAD moieties were not incorporated in place of adenosine as a noncanonical nucleotide during transcription initiation and strongly implicate the existence of a post-transcription initiation capping mechanism.

The best-characterized mechanism of NAD cap addition currently is the ab initio addition of non-canonical caps including NAD caps. This is primarily based on in vitro studies demonstrating that the RNA polymerases from both prokaryotes and eukaryotes can add a NAD cap in place of ATP at the 5′-end during transcription initiation^25^. An alternative mode of post-transcriptional addition of the NAD caps has also been proposed based on the presence of NAD caps on small nucleolar RNAs (snoRNAs) whose 5′ ends are generated by exoribonucleases and cannot undergo ab initio capping^10^. In the present study, we expand on these results and demonstrate in addition to snoRNAs, cytoplasmic mRNA can also be NAD-capped post-transcriptionally suggesting the presence of dedicated capping machinery at least in *S.cerevisiae*. The demonstration of both human and plant snoRNAs with NAD caps^10,33^ also suggests that post-transcriptional NAD capping is likely a general mechanism in eukaryotes. Our data is consistent with two modes of NAD cap addition, one being ab initio addition and the second through a yet uncharacterized NAD capping mechanism. Collectively, our findings reveal the complex nature of cellular NAD capping and the limitation of our current understanding of this intriguing modification.

The ability to more accurately identify NAD-RNAs enabled us to assess their presence on actively translating ribosomes and address whether NAD-RNAs can be translated. Consistent with our previous assessment that in vitro generated exogenous NAD-RNAs are not translated^10^ we demonstrated that nuclear-encoded NAD-RNAs minimally interact with a translationally competitive pool of cytoplasmic ribosomes and do not appear to be engaged in translation in yeast cells under normal growth conditions. Remarkably, a more dramatic outcome was detected in mitochondria. Mitochondrial protein-coding NAD-RNAs were detected to be engaged within the translationally competent pool of mitochondrial ribosomes, consistent with undergoing active translation. One possibility for the differing states of translatability between the cytoplasmic and mitochondrial transcripts could be the requirement of the m^7^G cap for the cytoplasmic translation^1^ but not mitochondrial translation^34^. However, the formal possibility that these NAD transcripts are not engaged in translation but function as regulatory RNAs cannot be ruled out. Future studies will address any potential role of NAD cap in mitochondrial translation.

## Methods

### Yeast growth and media

Yeast cells (BY4741 strain background) WT and xrn1-H41A^22^ were grown at 30 °C in (1% w/v yeast extract, 2% w/v peptone, 2% w/v glucose/glycerol). All yeast strains used in the present study are listed in Supplementary Table 2.

### In vitro transcription of NAD and m^7^G-capped RNAs

RNAs containing NAD and m^7^G cap structures were synthesized by in vitro transcription from synthetic double-stranded DNA template ɸ2.5-NAD-40, ɸ2.5-NAD-75 containing the T7 ɸ2.5 promoter, and a single adenosine within the transcript positioned at the transcription start site (Supplementary Table 3). For m^7^G-capped RNA, m^7^G-(5′) PPP (5′)-A RNA Cap Structure Analog (New England Biolabs) was included in the transcription reaction, whereas for NAD-RNA, NAD was used instead of ATP. In vitro transcription was carried out at 37 °C overnight, using HiScribe^TM^ T7 High yield RNA Synthesis kit (New England Biolabs (NEB)). Following in vitro transcription, RNA was purified using Monarch^®^RNA Cleanup Columns (NEB) as per the manufacturer’s instructions.

### RNA in vitro deNADding and decapping assays

For deNADding, the NAD-RNAs were incubated with 100 nM SpRai1 recombinant protein in NEB buffer (100 mM NaCl, 50 mM Tris-HCl, 10 mM MgCl_2_, pH 7.9). Reactions were incubated at 37 °C for 1 hour, and the deNADed RNA was purified using Monarch^®^RNA Cleanup Columns (NEB) as per the manufacturer’s instructions.

For decapping of both in vitro transcribed m^7^G capped RNA and polyA enriched RNA from yeast cells, yeast DcpS enzyme (yDcpS; NEB) which we referred to by the SGD nomenclature, Dcs1, was used. Typically, the reaction was performed in 50 µL volume using 4 units per µg of capped RNA following the manufacturer’s protocol. The decapped RNA was purified using Monarch^®^RNA Cleanup Columns (NEB) as per the manufacturer’s instructions.

### Total RNA extraction and polyA+RNA enrichment

Total RNA from yeast strains was isolated with the acidic hot phenol method^35^ and treated with DNase (Promega) according to the manufacturer’s protocol. Total RNA from human cells was isolated using TRIzol reagent (Thermo Fisher Scientific) according to the manufacturer’s protocol and treated with DNase as explained above to eliminate DNA. To enrich for poly-adenylated RNA, total RNA purified as above was used as the input for the Poly(A) Purist MAG Kit (Thermo Fisher Scientific) following the manufacturer’s protocol. We retrieved ~1.5 µg of polyA RNA from 100 µg of total yeast RNA.

### Cu(I)-catalyzed Azide–Alkyne Cycloaddition (CuAAC) and Strain-promoted Alkyne-Azide Cycloaddition (SPAAC) reactions

To biotinylate NAD-capped RNAs using CuAAC, 50 µg of total RNA was first incubated with 10 µL of 4-pentyn-1-ol (Sigma-Aldrich), 10 µL of (125 µg/mL) of Adenosine diphosphate-ribosylcyclase (ADPRC) in a 100 µL reaction containing 50 mM HEPES, 5 mM MgCl_2_ (pH 7.0), and 40 U of RNasin® Ribonuclease Inhibitor (Promega) at 37 °C for 1 h. After the ADPRC treatment, RNAs were incubated with 250 µM biotin-PEG3-azide, freshly mixed 1 mM CuSO_4_, 0.5 mM THPTA, 2 mM sodium ascorbate in a 100 µL reaction with 50 mM HEPES, 5 mM MgCl_2_ (pH 7.0), and 40 U of RNasin® Ribonuclease Inhibitor (Promega) at 30 °C for 30 min. The RNA was then precipitated with ethanol in the presence of 2 mM EDTA and 2 M ammonium acetate^10,36^. For reactions using the SPAAC approach, ADPRC was first used to replace the nicotinamide and add an azide residue onto the NAD-RNA. Typically, these reactions were performed in a 100 µL volume with either total RNA or polyA-purified RNA dissolved in DEPC water, 10% (v/v) of 3-azido-1-propanol (Sigma) together with 10 µL of (125 µg/mL) of ADPRC (Sigma) in ADPRC reaction buffer (50 mM HEPES (pH 7.0) and 5 mM MgCl_2_). The reaction was carried out at 37 °C for 1 hour. The transglycosylated RNA with the azide residues was next purified using Monarch^®^RNA Cleanup Columns (NEB) and eluted in 20 µL of DEPC water. For the SPAAC reaction, dibenzocyclooctyne-PEG4-biotin (DBCO-biotin, Sigma) was used as the alkyne moiety for the cycloaddition. Following the ADPRC reaction, both total RNA and polyA enriched RNA in pure water were mixed with 1x volumes of MK-Gel Loading Buffer (90% Formamide, 15 mM EDTA, and 0.025% SDS) and 500 μM DBCO-biotin (Sigma). Typically, these reactions were 20 μL MK-Gel Loading Buffer, 18 μL RNA (from ADPRC reaction), and 2 μL 10 mM stock of the DBCO reagent. The reactions were performed at 55 °C for 15 minutes to denature the RNA and stopped by adding 310 μL water followed by purification of the conjugated RNA using Monarch^®^RNA Cleanup Columns (NEB). The products were analyzed by gel electrophoresis or subjected to affinity purification as described below.

### RNA gel electrophoresis, capillary blotting, and near-InfraRed fluorescent imaging

For blotting analysis of either enriched RNA following biotin-mediated affinity purification or directly after the SPAAC reaction, RNA was incubated at 90 °C for 2 min in formaldehyde loading buffer (FLB) (50% Formamide, 6% formaldehyde, 50 mM HEPES (pH 7.8), 0.5 µg/mL ethidium bromide and 10% glycerol). Samples were then loaded onto a 1% agarose gel and resolved by electrophoreses at 120 V for 1 h. Before transferring the gel onto a 0.45 μm nitrocellulose membrane (Cytiva), total RNA was first visualized using a UV gel transilluminator. RNA was transferred overnight by passive, upward transfer facilitated by the capillary flow of the 10X SSC buffer. After transfer, RNA was cross-linked to the NC using UV-C light (0.2 J/cm2). The membrane was next blocked with Odyssey Blocking Buffer, PBS (Li-Cor Biosciences) for 30 minutes at room temperature (RT). After blocking, IRDye® 800CW streptavidin (Li-Cor Biosciences) was diluted to 1:7,000 in Odyssey Blocking Buffer and stained the NC membrane for 30 minutes at RT. The membrane was next washed three times with PBST (ResearchProductInternational). Before scanning, the membranes were briefly rinsed in 1x PBS and scanned by an Odyssey Fc (Li-Cor Biosciences) with the software prearranged to auto-detect the signal intensity for both the 600 and 800 channels. The 600 channel was used for ethidium bromide and the 800 channel was used for the detection of IRDye® 800CW streptavidin.

To assess the residual activity of ADPRC towards the m^7^GpppA capped RNA, we mimicked the in vivo differential by mixing in vitro transcribed 40 nts NAD-RNA and 75 nts m^7^GpppA capped RNA at molar ratios of ~50:1. The mixture was subjected to the SPAAC- reaction as discussed above with the only difference that instead of DBCO-biotin, DBCO-Cy3 was used for cycloaddition. The RNA was purified over the Monarch^®^RNA Cleanup Columns (NEB). The eluted RNA was next mixed with an equal amount of 2X MK- Gel Loading Buffer and denatured at 65 °C before resolving on a 7 M 15% PAGE gel. The ethidium bromide-stained RNA was first visualized in the gel using a UV gel transilluminator and the Cy3 conjugated RNA was detected using the Cy3 channel on a Typhoon scanner (GE)

### NADcapPro seq

Fifteen micrograms of polyA RNA were isolated from three independent biological replicates of both yeast WT and *xrn1-H41* mutant and were treated with Dcs1 as described above to remove m^7^G-caps. Samples without Dcs1 treatment (SPAAC) and ADPRC (minus ADPRC) were also processed similarly to assess the contribution from m^7^G caps and any background noise due to highly abundant RNAs. Following the SPAAC reactions, enrichment was achieved by selective affinity to streptavidin beads^10^. Briefly, RNAs were incubated at 25 °C for 30 min with 15 μL of magnetic Dynabeads™ MyOne™ Streptavidin T1, which were pre-blocked with 100 ng/µL of bacterial small RNAs in 100 μL of immobilization buffer containing 10 mM Tris-HCl (pH 7.5) 1 mM EDTA and 2 M NaCl. After rigorous washing with wash buffer (10 mM Urea, 5 mM Tris-HCl [pH 7.5], 0.5 mM EDTA, and 1 M NaCl) five times at 25 °C for 5 min each, biotinylated RNAs were eluted by incubating the beads with 20 μL of MK-Gel Loading Buffer at 90 °C for 2 min. The biotinylated RNAs were next purified using Monarch^®^RNA Cleanup Columns (NEB) as described above and eluted in 10 µL of nuclease-free water. The samples were next flash-frozen in liquid nitrogen and were sequenced at GENEWIZ (Azenta Life Sciences).

### NADcapPro sequencing analysis

Raw reads were processed for quality control and adapter removal by fastp^37^. Following this, in silico rRNA depletion was accomplished by using hisat2^38^ to align the reads to rRNA sequences and save the reads that did not align. Transcript abundances were quantified by aligning these rRNA-free files to a reference transcriptome (https://ftp.ncbi.nlm.nih.gov/genomes/all/GCF/000/146/045/GCF_000146045.2_R64/GCF_000146045.2_R64_rna.fna.gz) using Kallisto^39^. The resulting counts were rounded and TPMs recalculated based on these rounded counts. Read count distributions for rounded count can be seen in Supplementary Fig. 6a. Pairwise correlations based on the recalculated TPMs produced high correlations between replicates (R > 0.99, Supplementary Fig. 6b). Further analyses were based on these numbers and were performed using the R programming language version 4.2.1^40^ and the Tidyverse set of packages^41^. Differential expression was performed using DESeq2^23^ with the “apeglm” method of normalization^42^. The GO term analysis was performed using Gene Ontology Slim Term Mapper (https://www.yeastgenome.org/goSlimMapper). To determine if the number of overlaps observed in Fig. 3c, d are unique, we generated an expected value of overlaps by keeping the same number but randomizing the identity of significant genes in each category and then recomputing the number of overlapping genes 10,000 times. The resulting distribution of overlaps +/− 1 standard deviation was less than the number of observed overlaps in both comparisons (Fig. 3c and d). We used a chi-squared test to test for the independence of our categories in each comparison. This is now presented in the Supplementary Fig. 6c.

### circNC analysis

In total, 5 µg of yeast polyA RNA was first dephosphorylated using Quick CIP (NEB) to eliminate potential 5′ end phosphorylated RNA contamination in the subsequent reactions. Typically, these reactions were performed in a 100 µL volume with polyA-purified RNA dissolved in DEPC water, with 6 µL of (CIP) in 10X rCutSmart™ Buffer at 37 °C for 40 minutes. The dephosphorylated RNAs were next purified using Monarch^®^RNA Cleanup Columns (NEB) as described above and eluted in 30 µL of nuclease-free water. The dephosphorylated RNAs are next divided into 3 equal fractions of 10 µL each and treated separately with either no enzyme (Ctrl) or MDE (NEB) or Rai1. The MDE reaction was performed using NEB’s protocol at 37 °C for 1 hour, whereas the Rai1 reaction was performed in 50 µL volume with NEB buffer 3 and 0.5 µL RNAsin at 37 °C for 1 h. The reaction products were next purified using Monarch^®^RNA Cleanup Columns (NEB) and eluted in 15 µL DEPC water. All Ctrl, MDE, and Rai1 treated RNAs were next subjected to intramolecular ligation using T4 RNA ligase 1 (NEB). Typically, the ligation reaction was performed in a 50 µL volume with 15 µL of Ctrl, MDE, and Rai1 treated RNA following the manufacturer’s protocol in the presence of ~25% PEG8000 at 25 °C for 16 hours. The reaction products were next purified using Monarch^®^RNA Cleanup Columns (NEB) and eluted in 15 µL DEPC water and reverse transcribed with M-MLV reverse transcriptase and gene-specific primers with gene name -P1 in Fig. 4a (sequence listed in Supplementary Table 3) as per the manufacturer’s guidelines. PCR was performed with the primers (gene name-P2 and gene name-P3 in Fig. 4a) listed in Supplementary Table 3 with Phire Plant Direct PCR Master Mix (Thermo Scientific™). The PCR products were next resolved onto either 1.5 or 2% Agarose-TAE gel, sliced, purified, and sequenced using Amplicon-EZ (GENEWIZ (Azenta Life Sciences)) sequencing.

### circNC sequencing analysis

Raw reads were processed for quality control and adapter removal by fastp^37^. The analysis of this sequencing data was dependent on our PCR products spanning the junction between the 5′ and 3′ ends of a transcript. We chose primer locations near the start and end of the coding sequences for each gene (Fig. 4a). However, because the UTR lengths for many yeast genes are unknown and our read lengths are maximally ~500 bases (from 250 bp paired-end reads), it was unclear if read 1 or 2 individually, or both would cover the junction. To ensure that we were able to analyze the junction regardless of which read covered it, we considered read 1 and read 2 separately, as well as the merged reads. Reads were merged using BBmap’s bbmerge script^43^. Additionally, we expected that if a read covered our junction, it should contain a polyA tail^44^. We used cutadapt^45^ to filter our reads for those containing a stretch of at least 8 A’s in a row. After filtering was complete, we counted the unique reads in each file (read 1, read 2, and merge) individually and used the top 5 most abundant unique reads for multiple sequence alignments (using the R package msa^46^) which consisted of the top 5 from an MDE sample and from a Rai1 sample. Manual, visual analysis of the junction was carried out on the reads where the junction could be detected.

### Sucrose gradient centrifugation

For polysome fractionation, exponentially grown yeast cells in 500 mL YPD (OD _600nm_~2) were first treated with 100 µg/mL cycloheximide for 10 minutes at room temperature. The cells were next harvested, washed once in ice-cold 1x PBS, and lysed using mortar and pestle in liquid nitrogen. The cell powder obtained after grinding was resuspended in polysome buffer A (20-mM Tris-HCl pH7.4, 150-mM KCl, 3-mM MgCl2, 1-mM DTT, 0.5% v/v NP-40 and EDTA- free complete protease inhibitor, Roche)^47^ and incubated at 4 °C for 30 min. Samples were then centrifuged for 10 min at 10,000 × *g* to remove the cell debris. Approximately 40 A260 units were layered onto 10–50% w/v sucrose gradients prepared in buffer A. The gradients were made with Gradient Master 107 (Biocomp). These gradients were next centrifuged for 3 h at 39,000 rpm and 4 °C in a Beckman-Coulter ultra-centrifuge using the SW40 rotor. After centrifugation, fractions corresponding to each peak (Fig. 5a)–free pool, the 40 S, 60 S, 80 S, and polysomes were collected from the top of each gradient, by use of Biocomp Gradient Station. The absorbance at 254 nm was measured during the collection. Next, ribonucleoproteins from each fraction were precipitated using 1/10^th^ (v/v) 3 M sodium acetate (pH5.2) and 2X volume of 100% ethanol to get rid of sucrose. The ribonucleoprotein pellet was next resuspended in 400 µL of DEPC water and total RNA from each fraction was isolated using Trizol Reagent (Invitrogen) following manufacturers’ instructions. For circNC analysis, polyA RNA from each fraction was isolated as explained above.

### Isolation of translationally active mitochondrial ribosomes

Translationally active mitoribosomes were isolated as described before using C-terminally 3X FLAG-tagged MRPS17 protein as a bait^28^. MRPS17-FLAG strain was generated using homologous recombination using primers listed in Supplementary Table 3 and using plasmid pFA6a-6xGLY-3xFLAG-kanMX6 as a template. 5 g of exponentially grown yeast cell pellet (OD600 ~2) in YPEG (1% yeast extract, 2% Peptone, 2% ethanol, and 2% Glycerol) was harvested and flash frozen in liquid nitrogen. Cells were next lysed using a mortar and pestle in liquid nitrogen. The cell powder obtained after grinding was resuspended in a lysis buffer^28,48^ (10 mM Tris, pH 8.0, 50 mM NH4Cl, 10 mM MgCl_2_, 0.5% CHAPS, and 2X protease inhibitor cocktail (Complete, EDTA-free, Roche)) and incubated at 4 °C for 30 min. The lysate was next clarified by centrifugation for 10 min at 5000 × *g* to remove the cell debris. The clarified lysate was then transferred to a fresh 15 mL centrifuge tube and the protein concertation was assessed using a Bradford assay. 5 mg of protein lysate was added to the pre-equilibrated (in 1X lysis buffer) anti-Flag M2 magnetic beads (Sigma). The mixture was rotated end-over-end at 4 °C for 3 h. Next, the beads were washed 3X with wash buffer (10 mM Tris pH 8.0, 50 mM NH_4_Cl, 10 mM MgCl_2_, 0.1% CHAPS) for 5 min at 4 °C and the FLAG-tagged protein was eluted by incubation with 200 μg/mL Flag peptide (Sigma). The eluate was next assessed for the presence of MRPS17 by Western Blotting using anti-FLAG antibody (Sigma) (1;10,000). RNA from the eluate was isolated using Phenol-Chloroform as described above.

### Boronate affinity electrophoresis and Northern Blot analysis of in vivo NAD capped transcripts after DNAzyme mediated RNA cleavage

Boronate affinity electrophoresis was performed as described recently^22^. 30 μg of total cellular RNA or the RNA retrieved from the mitoribosomes eluates (as described above) were incubated with 1 μM of the corresponding DNAzyme in a 50 μL reaction containing 10 mM Tris-HCl pH = 8.0, 50 mM NaCl, 2 mM DTT, and 10 mM MgCl_2_. Samples were denatured at 85 °C for 2 min and gradually cooled to 37 °C. MgCl_2_ was added to a final concentration of 10 mM and incubated for 60 min at 37 °C. Reactions were stopped with 100 μL of stop solution (50 mM Tris pH 8.0, 20 mM EDTA, and 0.1 µg/mL glycogen), and RNA was precipitated with 500 μL ethanol by incubating for 30 min at –80 °C followed by centrifugation for 30 min at 16,000 × *g* at 4 °C. The supernatant was removed, and the pellet was resuspended in H_2_O.

NAD-capping was analyzed using DNAzyme-generated fragments of mitochondrial RNAs. The cleaved RNA was resolved on 8% urea polyacrylamide gels supplemented with 0.3% 3-acrylamidophenylboronic acid (Boron Molecular). RNA was next transferred to a positively charged Nylon transfer membrane (GE Healthcare Life Sciences) and incubated with a ^32^P-labeled DNA probe complementary to the 5′-end fragments of target RNAs (Supplementary Table 3). The probes were labeled using T4 polynucleotide kinase (NEB) and [γ-^32^P] ATP (Perkin Elmer). Reaction products were visualized with Amersham Typhoon RGB Biomolecular Imager (GE Healthcare Life Sciences).

### Statistics and reproducibility

The present study utilized biological triplicates for all samples in the NAD CapPro Seq and regular RNA seq experiments to ensure statistical robustness and reproducibility. Statistical analysis was conducted using Student’s t-test (single-tail), and chi-squared test to assess the independence of categories in each comparison. The analyses were performed using the Tidyverse package and other general statistics packages of the R programming language version 4.2.1. The code used is available at https://github.com/shahlab/NADcapPro. Differential expression analysis was conducted as described above using DESeq2 with the “apeglm” as the method of normalization to control for potential biases and improve the accuracy of the results.

### Reporting summary

Further information on research design is available in the Nature Portfolio Reporting Summary linked to this article.

## Supplementary information

Supplementary Material

Description of Additional Supplementary Files

Supplementary Data 1

Reporting Summary

## Data Availability

All unique materials and reagents generated in this study are available from the corresponding author with a completed material transfer agreement. The sequencing data is deposited at GSE217259. The code for analyses is available at https://github.com/shahlab/NADcapPro. Any additional information required to reanalyze the data reported in this paper is available from the corresponding author upon request. All data are available in the main text or the supplementary materials.
