# Supplementary material for: NADcapPro and circNC: methods for accurate profiling of NAD and non-canonical RNA caps in eukaryotes
Source: Commun Biol. 2023 Apr 13;6:406. doi: 10.1038/s42003-023-04774-6 (PMC10101982; doi:10.1038/s42003-023-04774-6)
Supplement: Supplementary file 1 — Supplementary Material [file 42003_2023_4774_MOESM1_ESM.pdf]

NADcapPro and circNC: Methods for Accurate Profiling of NAD and Non-Canonical RNA Caps in Eukaryotes

Sunny Sharma[1], Jun Yang[1], John Favate[2], Premal Shah[2], and Megerditch Kiledjian[1*]

[1] Department of Cell Biology and Neurosciences, Rutgers University, Piscataway, NJ-08854
[2] Department of Genetics, Rutgers University, Piscataway, NJ-08854
[*]Corresponding author: kiledjian@biology.rutgers.edu

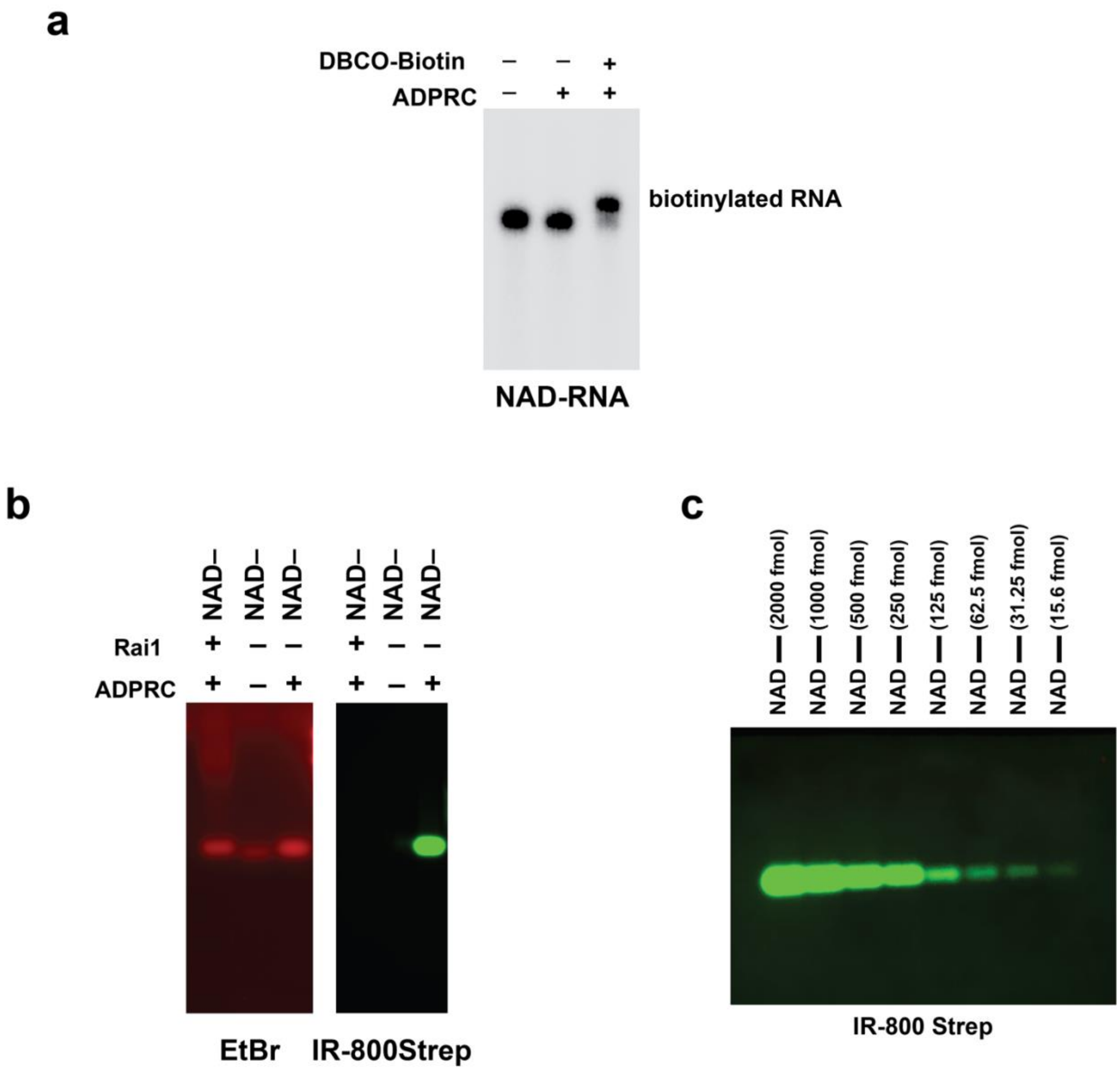

Supplementary Figure 1. Sensitivity of IR dye for the detection of NAD-capped RNAs. (a) Efficiency of SPAAC reaction in biotinylating the NAD capped RNAs post ADPRC treatment. **(b)**Ten picomoles of 40 nts *in vitro* transcribed NAD-capped RNA and its deNADded form (Rai1 treated) were subjected to the SPAAC reaction and visualized using IR dye. **(c)** Different concentrations of an *in vitro* transcribed 40nts NAD-capped RNA were subjected to the SPAAC reaction and biotin-conjugated RNAs were detected by the IR dye at levels as low as 15 fmol

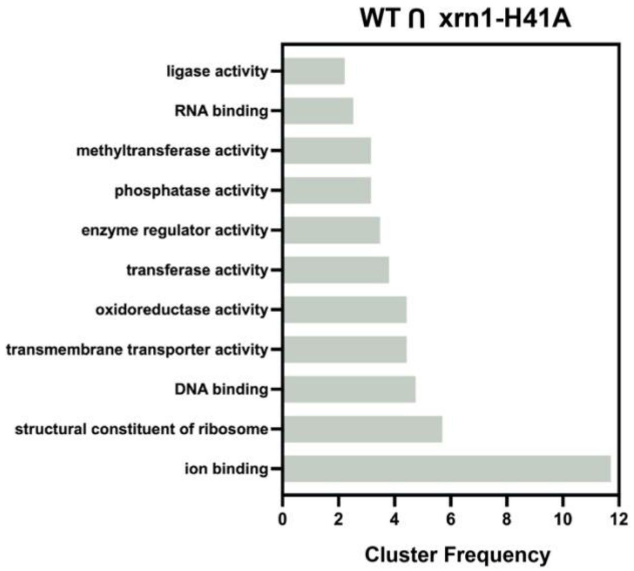

Supplementary Figure 2. Gene Ontology analysis. Gene ontology categories for the NAD-capped gene sets common in WT and xrn1-H41A knockin mutant.

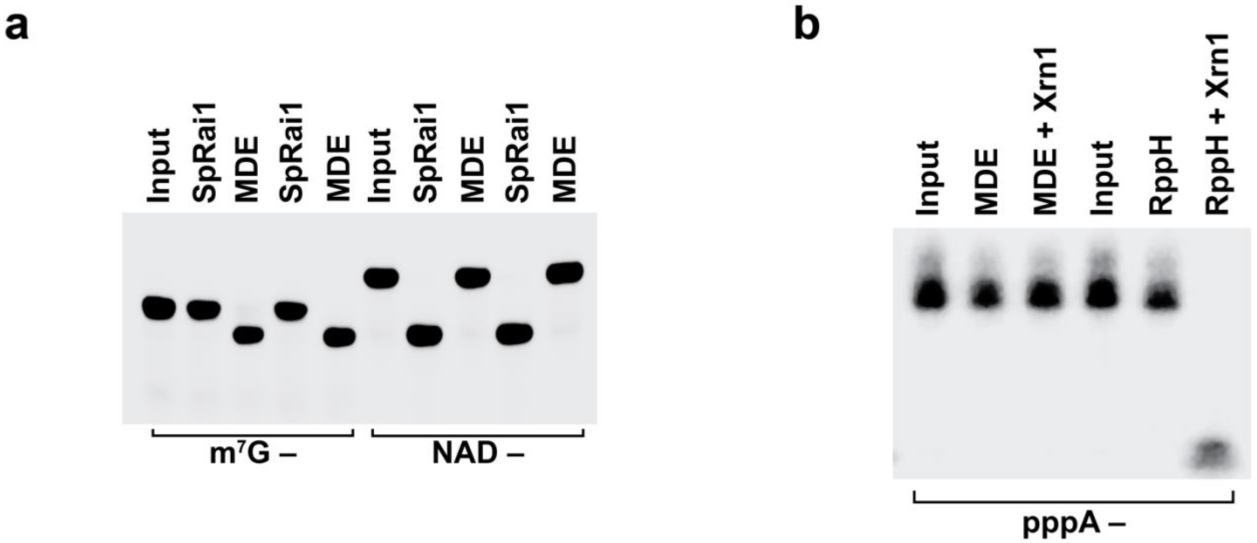

Supplementary Figure 3. Substrate specificity of MDE and Rai1. **(a)** Uniformly ^{32}P-labeled m^{7}G or NAD-capped RNA (40nts) RNA were treated with Rai1 and MDE and the reaction products were resolved onto a 7M Urea 8%PAGE, 0.2% APB TAE gel. **(b)** To evaluate the activity of MDE towards pppA– RNA, uniformly ^{32}P-labeled RNA containing pppA (triphosphate) (40 nucleotides) was treated with either MDE or MDE plus Xrn1. The same RNA sample was also subjected to RppH reaction as a control.

a

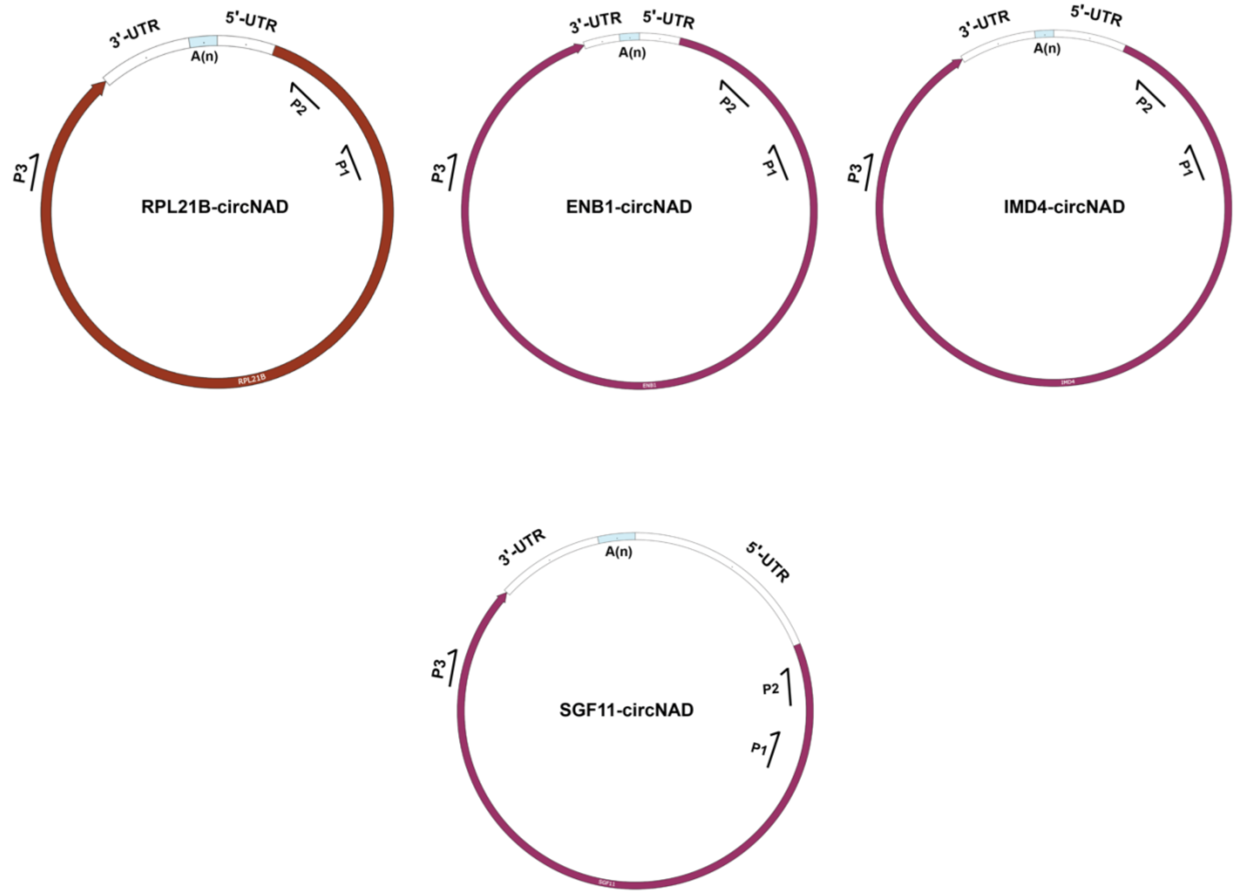

b

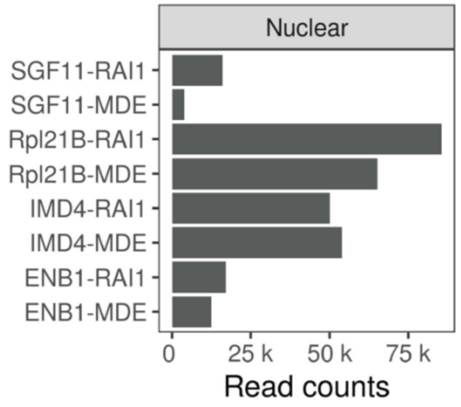

Supplementary Figure 4. circNC analysis (a) Schematic illustration of the circularised transcripts with the locations of the respective primers (P1, P2, and P3) used to make PCR shown in Fig. 4b. **(b)** Number of read counts used to assess the site of NAD cap addition (Fig. 4c)

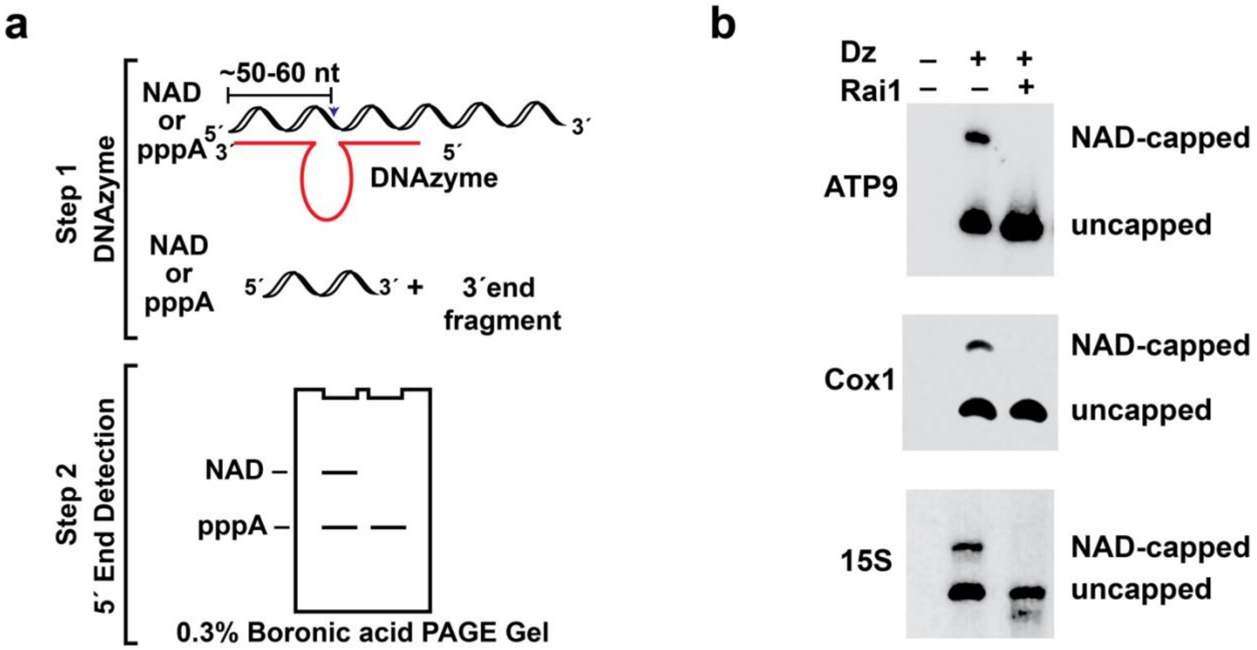

Supplementary Figure 5. Detection of NAD-capped mitochondrial RNA *in vivo* by DNAzyme and boronate affinity electrophoresis (a) Schematic representation of DNAzyme-mediated RNA cleavage coupled to Northern Blot detection of NAD-capped RNAs **(b)** Northern Blot analysis of transcript-specific DNAzyme-generated 5′-end-containing subfragments of the total RNA resolved on 0.3% 3-acrylamidophenylboronic acid 10% PAGE gel and detected with the ^{32}P labeled transcript-specific probes of the indicated RNAs.

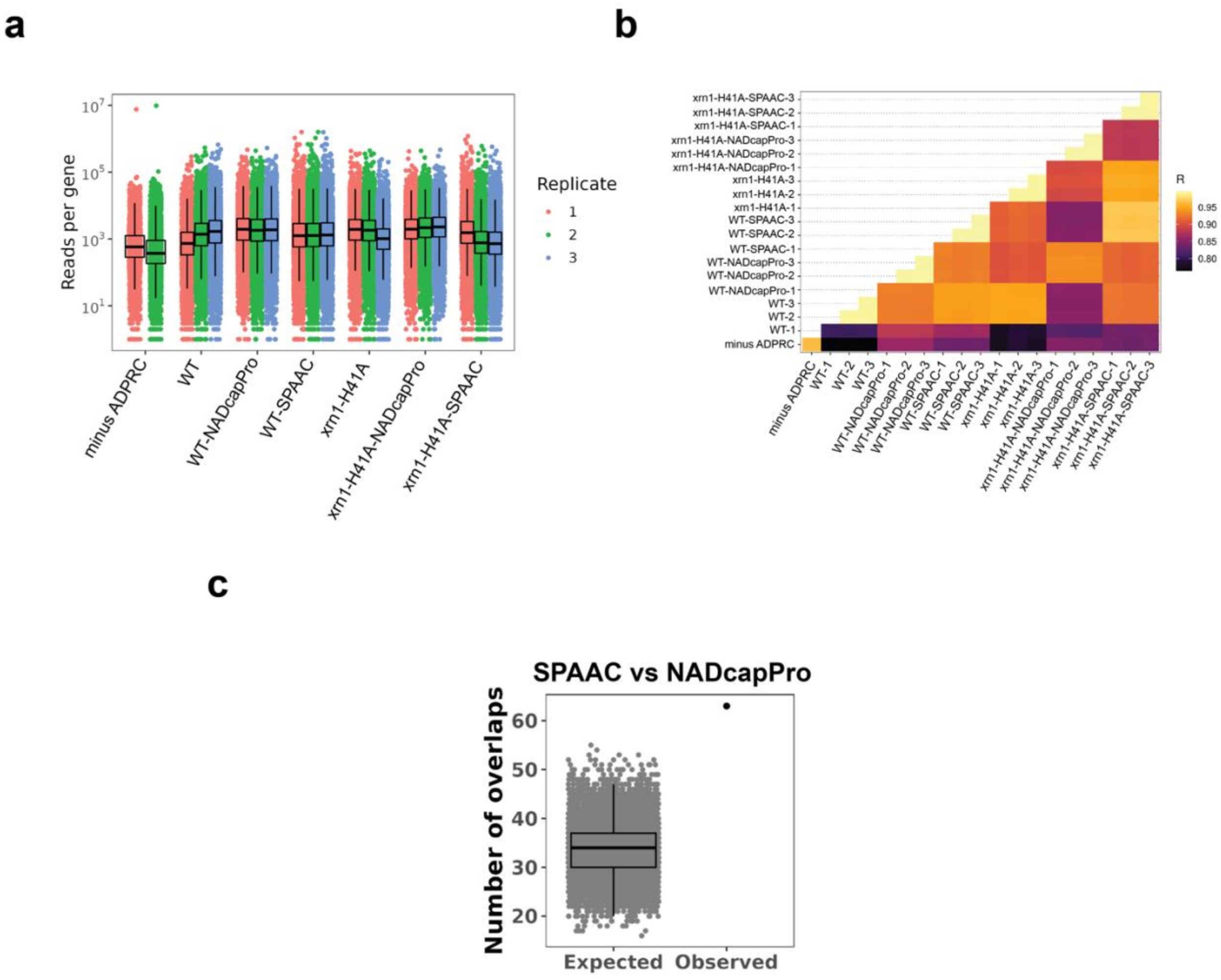

	NS	Sig
SPAAC seq (WT)	5838	268
NADcapPro seq (WT)	5337	769

$X^2 = 263.5$
p = 3.00 e-59

NS– Not Signficant
Sig– Signficant

Supplementary Figure 6. NADcapPro Seq analysis of RNAs isolated from yeast WT, and xrn1-H41A cells (a) The distributions of read counts per gene in each replicate. **(b)** Pairwise correlation based on TPMs **(c)** Calculation of the significance of the overlap observed between SPAAC vs NADcapPro and WT(figure 3c). To determine if the number of overlaps we observed is exceptional, we generated an expected value of overlaps by keeping the same number but randomizing the identity of significant genes in each category and then recomputing the number of overlapping genes 10,000 times. The resulting distribution of overlaps+/- 1 standard deviation was less than the number of observed overlaps in both comparisons. A chi-squared tes (X^2) was used to test for the independence of our categories in each comparison and is presented below for each comparison.

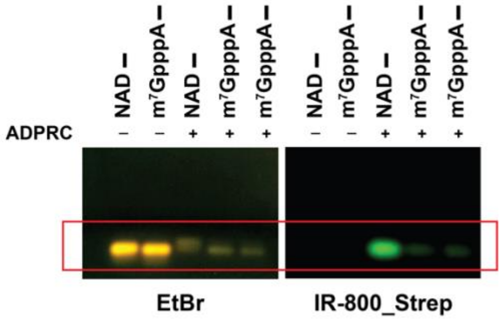

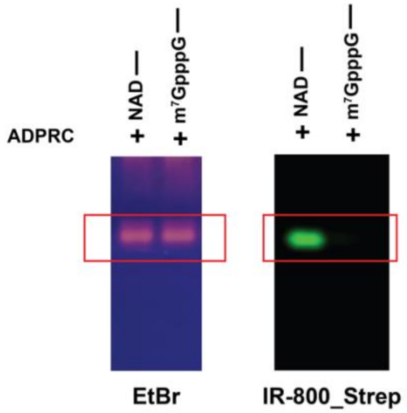

Supplementary Figure 7. Uncropped gels and immuno-blots for figure 2a.

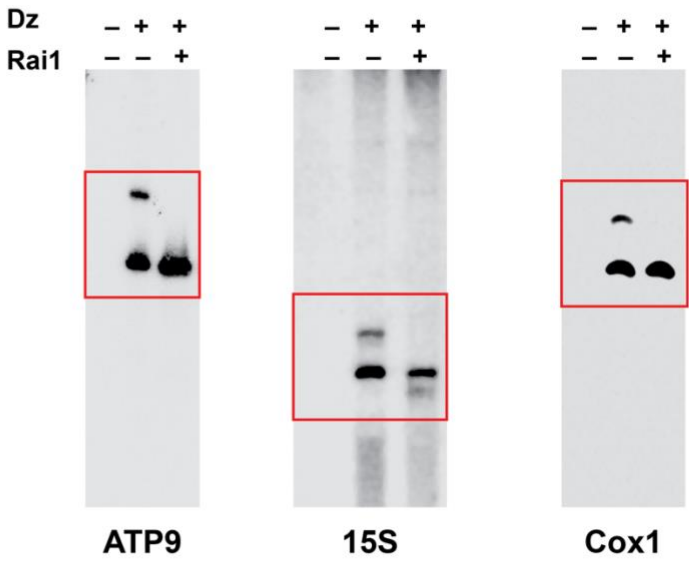

Supplementary Figure 8. Uncropped Northern blots for the supplementary figure 5b.

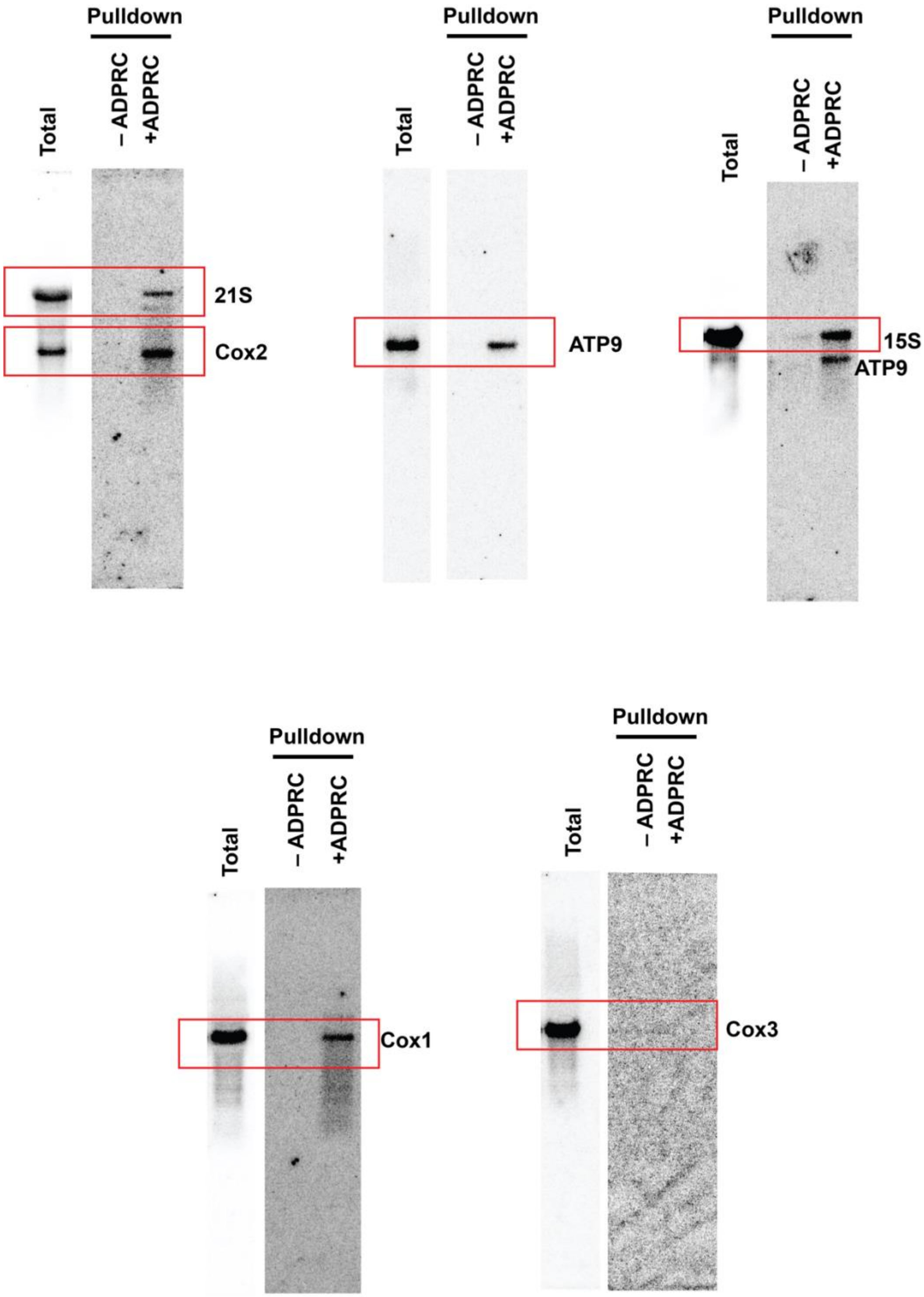

Supplementary Figure 9. Uncropped Northern blots for supplementary figure 6a.

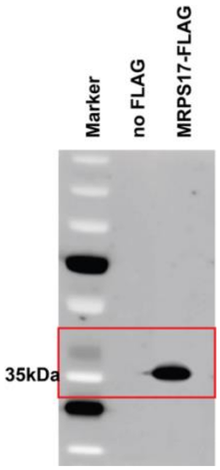

Supplementary Figure 10. Uncropped Western blot for figure 6d.

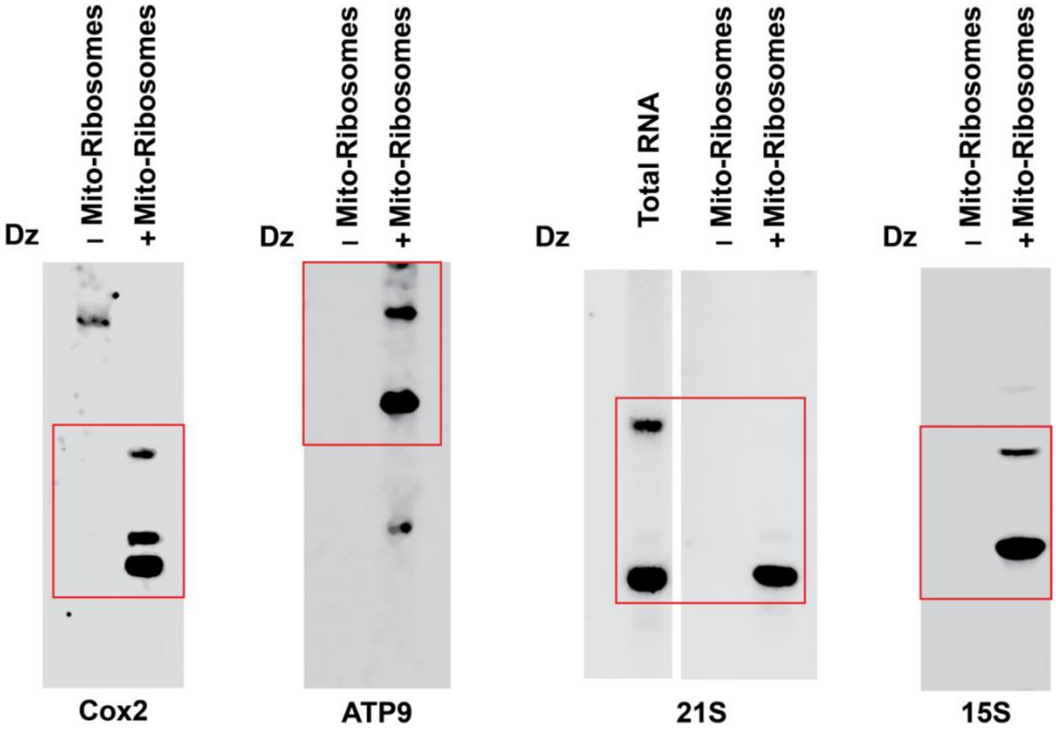

Supplementary Figure 11. Uncropped Western blot for figure 6f and g.

Supplementary Table 1: List of NAD-capped RNAs

GO Term :Transposition

	**ANNOTATED GENES**
**1**	YAR010C
**2**	**YBL100W**-A
**3**	YBR012W-A
**4**	YDR034C-C
**5**	YDR098C-A
**6**	YDR098C-B
**7**	YDR170W-A
**8**	YDR210C-C
**9**	YDR210W-A
**10**	YDR261W-A
**11**	YDR316W-A
**12**	YER159C-A
**13**	YFL002W-B
**14**	YGR027W-A
15	YGR038C-A
**16**	**YGR109W-A**
**17**	YGR109W-B
**18**	YGR161C-C
**19**	\YGR161W-A
**20**	YHR214C-C
**21**	YJR026W
**22**	YLR227W-A
**23**	YLR256W-A
**24**	YLR410W-A
**25**	YML040W
**26**	YML045W-A
**27**	YMR051C
**28**	YNL054W-A
**29**	YNL284C-A
**30**	YOR142W-A
**31**	YOR192C-A
**31**	YOR343W-A
**32**	YPR137C-A
**33**	YPR158C-C
**34**	YPR158W-A

Supplementary Table 2. Yeast strains and plasmids used in the present study.

**Yeast Strains /Plasmids**	**Source**	**Identifier**
BY4741 (MATa; his3Δ1; leu2Δ0; met15Δ0; ura3Δ0)	Dharmacon	YSC1048
xrn1-H41A (BY4741; MATa; his3Δ1; leu2Δ0; met15Δ0; ura3Δ0; xrn1(C121G,A122C))	(*1*)	NA
MRPS17-FLAG (BY4741; MATa; his3Δ1; leu2Δ0; met15Δ0; ura3Δ0; MRPS17*::FLAG-kanMX4; MRPS17::3xFLAG-kanMX4*)	This study	NA
pFA6a-6xGLY-3xFLAG-kanMX6	addgene	#20754

Supplementary Table 3. Oligonucleotides used in the present study

**Name**	**Sequence**
ϕ2.5-NAD-40-	CAGTAATACGACTCACTATTAGTTGGTGGTTGTTGTGTGTTTGTGGTTGGTTTGTT TGGC (*1*)
ϕ2.5-NAD-75-	CAGTAATACGACTCACTATTAGTTGGTGGTTGTTGTGTGTTTGTGGTTGGTTTGTT TGGCGGGGTCTCTCTTCTCCCTCTCTCTCTTTCTTCTTG
IVT-G-40 -	TAATACGACTCACTATAGGGatctcccaatcccctataatcaactctcaaccacacgcg
15S-Dz-	tttacttttccttattaGGCTAGCTACAACGAataactaatataataatatatat
21S-Dz-	ACTCCATGATTAGGCTAGCTACAACGACTCTTTAAATCT
ATP9-Dz-	cattatattaataatatatatatataGGCTAGCTACAACGAttatataaatgtgtataag
COX1-Dz-	atatatttaatgatattaataggctagctacaacgatctctttcataatct
COX2-Dz-	TCTTAATAAATCTAAGGCTAGCTACAACGAATTTTAATAAATCTT
COX3-Dz-	ttatatatatttttattatttaGGCTAGCTACAACGAatttattattaatataaatc
15S-probe-	atatatatatatatattattattattatataaaac
21S-probe-	ctatataataaatatttcaaatctattattctac
ATP9-probe-	gtgtataagatttatataattattattatattatg
COX1-probe-	ctatattattaatattatttatataaaatattaataa
COX2-probe-t	atcttaacctttagactcttttgtctatttataatatg
COX3-probe-	ttattaatataaatcattgataatatcttc
RPL21B-P1-	ACCTTGTAGATCTTCAAGTAGGTG
RPL21B-P2-	GGCACCATGCTTTCTGAAG
RPL21B-P3-	CGTATTGTCTCTACTGAAGGTAACG
ENB1-P1-	CCAGACGTGGTAGACTTCTC
ENB1-P2-	GTCTTTTCGCTGTAGCAGAC
ENB1-P3-	GAGAGAAAGAGAAGCTCAAAATC
IMD4-P1-	CTAAAGCCTTTTTGTAATCCAATG
IMD4-P2-	CCTTTGGTTCAAGCAGCAAAG
MRPS17-FLAG FP-	AAGAAAGCACGTCATGATGGATATGCAACAACCAAGCCAGgggggaggcgggggtggaga
MRPS17-FLAG RP-	GTTGAGAAAATATGACACTCGGCTTTCCTCTTTCTTTTATgaattcgagctcgtttaaac
