# Supplementary material for: NADcapPro and circNC: methods for accurate profiling of NAD and non-canonical RNA caps in eukaryotes
Source: Commun Biol. 2023 Apr 13;6:406. doi: 10.1038/s42003-023-04774-6 (PMC10101982; doi:10.1038/s42003-023-04774-6)
Supplement: Supplementary file 2 — Description of Additional Supplementary Files [file 42003_2023_4774_MOESM2_ESM.pdf]

Description of Additional Supplementary Files

File name: Supplementary Data 1

Description: circNC Junction Sequences.
