# Supplementary material for: NADcapPro and circNC: methods for accurate profiling of NAD and non-canonical RNA caps in eukaryotes
Source: Commun Biol. 2023 Apr 13;6:406. doi: 10.1038/s42003-023-04774-6 (PMC10101982; doi:10.1038/s42003-023-04774-6)
Supplement: Supplementary file 3 — Supplementary Data 1 [file 42003_2023_4774_MOESM3_ESM.pdf]

Gene	m^7G-capped<br>3'UTR–**(AA)n**–5'-UTR	% of transcripts	NAD-capped<br>3'UTR–**(AA)n**–5'-UTR	% of transcripts	Comments
**ENB1**	ATAATATTGTTGTCTGAAAT**AAAAAAAAAAAAAA**GCTCGTGAATGTCTCTCTGA	~90%	ATAATATTGTTGTCTGAAAT**AAAAAAAAAAAA**GGGTCCTAGCAGAACTCTAAT	~60%	
**IMD4-a**	TAAAAGTTTATTTTTGCATAT**AAAAAAAAAAAAA**GGCTTTTACATTTTCTCTGG	~53%	TCAACAAAACTAAAATAAAG**AAAAAAAAA**CATTTTACGATGAGTGCTG	~90%	**NAD capped transcripts contain longer (79nts) 3'UTRs than m7G capped transcripts (40nts)**
**IMD4-b**	TAAAAGTTTATTTTTGCATAT**AAAAAAAAAAAAA**GGATATTGGACCAATTCCAT	~40%			
**RPL21b**	ATAACAATTTACATGATATT**AAAAAAAAAAAAAAAA**GAGACAGTCAAGGACACTAA	~90%	ATAACAATTTACATGATATT**AAAAAAAAAAAAAAA**CTGAGACAGTCAAGGACACT	~75%	
**SGF11**	TTTATGCGGCATATCTGCAC**AAAAAAAAAAAA**CTTAGCGTTTCTTCCGTTGA	~72%	AACAATGCATTCAATTTTCT**AAAAAAAAAAAA**TATAGATTCTATTTCAAATG	~68%	**NAD capped transcripts contain longer(295nts) 3'UTRs than m7G capped transcripts (117nts)**
