# Supplementary material for: NADcapPro and circNC: methods for accurate profiling of NAD and non-canonical RNA caps in eukaryotes
Source: Commun Biol. 2023 Apr 13;6:406. doi: 10.1038/s42003-023-04774-6 (PMC10101982; doi:10.1038/s42003-023-04774-6)
Supplement: Supplementary file 4 — Reporting Summary [file 42003_2023_4774_MOESM4_ESM.pdf]

nature research

Corresponding author(s): Megerditch Kiledjian

Last updated by author(s): Mar 14, 2023

Reporting Summary

Nature Research wishes to improve the reproducibility of the work that we publish. This form provides structure for consistency and transparency in reporting. For further information on Nature Research policies, see Authors & Referees and the Editorial Policy Checklist.

Statistics

For all statistical analyses, confirm that the following items are present in the figure legend, table legend, main text, or Methods section.

n/a	Confirmed	
☐	☒	The exact sample size (*n*) for each experimental group/condition, given as a discrete number and unit of measurement
☐	☒	A statement on whether measurements were taken from distinct samples or whether the same sample was measured repeatedly
☐	☒	The statistical test(s) used AND whether they are one- or two-sided<br>*Only common tests should be described solely by name; describe more complex techniques in the Methods section.*
☒	☐	A description of all covariates tested
☒	☐	A description of any assumptions or corrections, such as tests of normality and adjustment for multiple comparisons
☐	☒	A full description of the statistical parameters including central tendency (e.g. means) or other basic estimates (e.g. regression coefficient) AND variation (e.g. standard deviation) or associated estimates of uncertainty (e.g. confidence intervals)
☐	☒	For null hypothesis testing, the test statistic (e.g. *F*, *t*, *r*) with confidence intervals, effect sizes, degrees of freedom and *P* value noted<br>*Give P values as exact values whenever suitable.*
☒	☐	For Bayesian analysis, information on the choice of priors and Markov chain Monte Carlo settings
☒	☐	For hierarchical and complex designs, identification of the appropriate level for tests and full reporting of outcomes
☒	☐	Estimates of effect sizes (e.g. Cohen's *d*, Pearson's *r*), indicating how they were calculated

Our web collection on statistics for biologists contains articles on many of the points above.

Software and code

Policy information about availability of computer code

Data collection	Code for analyses is available at https://github.com/shahlab/NADcapPro.
Data analysis	Raw reads were processed for quality control and adapter removal by fastp (Chen, S., Zhou, Y., Chen, Y. & Gu, J. fastp: an ultra-fast all-in-one FASTQ preprocessor. Bioinformatics 34, i884-i890 (2018). https://doi.org:10.1093/bioinformatics/bty560). Following this, in silico rRNA depletion was accomplished by using hisat2(Kim, D., Paggi, J. M., Park, C., Bennett, C. & Salzberg, S. L. Graph-based genome alignment and genotyping with HISAT2 and HISAT-genotype. Nat Biotechnol 37, 907-915 (2019). https://doi.org:10.1038/s41587-019-0201-4) to align the reads to rRNA sequences and save the reads that did not align. Transcript abundances were quantified by aligning these rRNA-free files to a reference transcriptome (https://ftp.ncbi.nlm.nih.gov/genomes/all/GCF/000/146/045/GCF_000146045.2_R64/GCF_000146045.2_R64_rna.fna.gz) using Kallisto (Bray, N. L., Pimentel, H., Melsted, P. & Pachter, L. Near-optimal probabilistic RNA-seq quantification. Nat Biotechnol 34, 525-527 (2016). https://doi.org:10.1038/nbt.3519) . The resulting counts were rounded and TPMs recalculated based on these rounded counts. Pairwise correlations based on the recalculated TPMs produced high correlations between replicates (R > 0.99). Further analyses were based on these numbers and were performed using the R (R , C., Team. R: A language and environment for statistical computing. R Foundation for Statistical Computing (2022)) programming language version 4.2.138 and the Tidyverse (Hadley Wickham, M. A., Jennifer Bryan, Winston Chang, Lucy D'Agostino McGowan, Romain François, Garrett Grolemund, Alex Hayes, Lionel Henry, Jim Hester, Max Kuhn, Thomas Lin Pedersen, Evan Miller1, Stephan Milton Bache, Kirill Müller, Jeroen Ooms, David Robinson, Dana Paige Seidel, Vitalie Spinu, Kohske Takahashi, Davis Vaughan, Claus Wilke, Kara Woo, and Hiroaki Yutani. Welcome to the Tidyverse. The Journal of Open Source Software 4, 1686 (2019). https://doi.org:https://doi.org/10.21105/joss.01686)set of packages . Differential expression was performed using DESeq2 22 with the "apeglm"(Zhu, A., Ibrahim, J. G. & Love, M. I. Heavy-tailed prior distributions for sequence count data: removing the noise and preserving large differences. Bioinformatics 35, 2084-2092 (2019). https://doi.org:10.1093/bioinformatics/bty895) method of normalization .

For manuscripts utilizing custom algorithms or software that are central to the research but not yet described in published literature, software must be made available to editors/reviewers. We strongly encourage code deposition in a community repository (e.g. GitHub). See the Nature Research guidelines for submitting code & software for further information.

Data

Policy information about availability of data

All manuscripts must include a data availability statement. This statement should provide the following information, where applicable:

- Accession codes, unique identifiers, or web links for publicly available datasets
- A list of figures that have associated raw data
- A description of any restrictions on data availability

The sequencing data is deposited at GSE217259. Reviewer token: wzypeqkuflgxvml.

Field-specific reporting

Please select the one below that is the best fit for your research. If you are not sure, read the appropriate sections before making your selection.

☒ Life sciences ☐ Behavioural & social sciences ☐ Ecological, evolutionary & environmental sciences

For a reference copy of the document with all sections, see nature.com/documents/nr-reporting-summary-flat.pdf

Life sciences study design

All studies must disclose on these points even when the disclosure is negative.

Sample size	The sample size was chosen from past knowledge on the good sample size to ensure adequate power. Sample size are indicated in figure legends.
Data exclusions	No data exclusions
Replication	Each experiment was performed with at least three independent biological replicates. All experimental results were derived from all experiments.
Randomization	No randomization was performed due to low sample number and the lack of its influence on the experimental design and output.
Blinding	Experiments required previous empirical knowledge for interpretation.

Reporting for specific materials, systems and methods

We require information from authors about some types of materials, experimental systems and methods used in many studies. Here, indicate whether each material, system or method listed is relevant to your study. If you are not sure if a list item applies to your research, read the appropriate section before selecting a response.

Materials & experimental systems

n/a	Involved in the study	
☐	☒	Antibodies
☒	☐	Eukaryotic cell lines
☒	☐	Palaeontology
☒	☐	Animals and other organisms
☒	☐	Human research participants
☒	☐	Clinical data

Methods

n/a	Involved in the study	
☒	☐	ChIP-seq
☒	☐	Flow cytometry
☒	☐	MRI-based neuroimaging

Antibodies

Antibodies used	Commercially available Monoclonal ANTI-FLAG® M2 antibody produced in mouse (F1804) from Milllipore Sigma.
Validation	Validations are carried out by the manufacturer(https://www.sigmaaldrich.com/deepweb/assets/sigmaaldrich/product/documents/144/194/vol6_iss2_antiflag_m2.pdf)<br>The ANTI-FLAG M2 mouse, affinity purified monoclonal antibody binds to fusion proteins containing a FLAG peptide sequence. The antibody recognizes the FLAG peptide sequence at the N-terminus, Met-N-terminus,C-terminus, and internal sites of the fusion protein.<br>Method of purification - Affinity tag purification.
